# Developments in Rapid Detection Methods for the Detection of Foodborne *Campylobacter* in the United States

**DOI:** 10.3389/fmicb.2018.03280

**Published:** 2019-01-23

**Authors:** Steven C. Ricke, Kristina M. Feye, W. Evan Chaney, Zhaohao Shi, Hilary Pavlidis, Yichao Yang

**Affiliations:** ^1^Department of Food Science, Center of Food Safety, University of Arkansas, Fayetteville, AR, United States; ^2^Diamond V, Cedar Rapids, IA, United States; ^3^Department of Poultry Science, University of Arkansas, Fayetteville, AR, United States

**Keywords:** *Campylobacter*, rapid detection, characterization, poultry, molecular technology

## Abstract

The accurate and rapid detection of *Campylobacter* spp. is critical for optimal surveillance throughout poultry processing in the United States. The further development of highly specific and sensitive assays to detect *Campylobacter* in poultry matrices has tremendous utility and potential for aiding the reduction of foodborne illness. The introduction and development of molecular methods such as polymerase chain reaction (PCR) have enhanced the diagnostic capabilities of the food industry to identify the presence of foodborne pathogens throughout poultry production. Further innovations in various methodologies, such as immune-based typing and detection as well as high throughput analyses, will provide important epidemiological data such as the identification of unique or region-specific *Campylobacter*. Comparable to traditional microbiology and enrichment techniques, molecular techniques/methods have the potential to have improved sensitivity and specificity, as well as speed of data acquisition. This review will focus on the development and application of rapid molecular methods for identifying and quantifying *Campylobacter* in U.S. poultry and the emergence of novel methods that are faster and more precise than traditional microbiological techniques.

## Introduction

*Campylobacter* species *Campylobacter jejuni* and *Campylobacter coli* are etiological agents of campylobacteriosis, which is a significant foodborne disease in the United States (Humphrey et al., 2007; Horrocks et al., 2009; Bolton, 2015). The Centers for Disease Control and Prevention (CDC) estimates that approximately 9% of foodborne illnesses in the United States originate from *Campylobacter* spp., with 15% of campylobacteriosis cases leading to hospitalization (Scallan et al., 2011). In recent years, despite other reservoirs for campylobacteriosis, chicken and other poultry products remain the primary contributors to campylobacteriosis. *Campylobacter* is not generally pathogenic in adult birds and is considered a commensal microorganism of the gastrointestinal microbiota; therefore, once *Campylobacter* successfully colonizes a few birds, it rapidly asymptomatically disseminates throughout the flock and is extremely difficult to track (Horrocks et al., 2009).

As a result, the status of *Campylobacter* in the bird is a significant concern to poultry producers as colonized birds often reach the processing plant undetected and lead to foodborne illness. As the demand for more poultry meat and faster line speeds increases in the United States, it will become important to develop rapid and reliable detection methods that enable real-time decision making for producers. Unfortunately, the poultry industry still relies on traditional microbiology-based approaches, which are time consuming and have relatively high limits of detection. In order to understand the relationship between traditional strategies to monitor for *Campylobacter* and the poultry industry, a critical review of the available culture methods in animal production, and specifically poultry production in the United States, was written by Huang et al. (2015). Huang and colleagues highlighted numerous issues associated with traditional culturing methods, such as media bias and time-to-data acquisition; however, the authors did not discuss alternative methods in depth (Huang et al., 2015). Non-culture-based methods should alleviate the concerns associated with microbiological approaches and enable the rapid assessment of the prevalence and even the absolute quantification of *Campylobacter* in poultry matrices. This review will focus on rapid, non-commercial molecular methods for the detection of *Campylobacter* and its potential use in U.S. poultry production systems.

## Challenges to the Identification and Detection of *Campylobacter*

*Campylobacter jejuni* and *C. coli* have the highest rate of foodborne-related clinical campylobacteriosis, and as a result, the detection and identification of *Campylobacter* are dedicated almost exclusively to these two species. Traditional microbiological culture methods have evolved over time to include the use of selective media and the optimization of growth conditions and antibiotic support to reduce co-cultured species of microorganisms. Media use guidelines necessitate the elevated incubation temperature (42°C) and a microaerophilic atmosphere to favor the growth of the thermophilic *Campylobacter*. Additionally, several antibiotics can be employed that repress the growth of non-*Campylobacter* species while simultaneously supporting the growth of naturally antibiotic-resistant isolates of *Campylobacter* (Eberle and Kiess, 2012). Once successfully isolated from poultry matrices, the further confirmation of the identification of *Campylobacter* includes phenotypic differentiation such as biotyping, serotyping, and multilocus enzyme electrophoresis discussed in detail by Eberle and Kiess (2012).

Despite the continued refined options for the isolation of *Campylobacter*, challenges remain, which reduce the efficiency of these methodologies. Unlike other foodborne pathogens like *Salmonella*, *Campylobacter* exhibits dynamic and malleable physiological and metabolic biological characteristics that can actively interfere with the sensitivity and specificity of culture-dependent methods. Evidence for this plasticity emerged during the assessment of multiple metabolic traits using an extensive panel of biochemical tests to evaluate multiple *Campylobacter* species (On et al., 1996). Data revealed significant and unique phenotypic diversity (On et al., 1996). This metabolic fluidity is further supported by evidence suggesting extensive genetic diversity and genomic instability in *C. jejuni* poultry isolates that may be environmentally influenced (Wassenaar et al. 1998; Wilson et al., 2010). Another good example of this plasticity is that when *Campylobacter* becomes stressed when exposed to psychrotrophic conditions during refrigeration and freezing, *Campylobacter* becomes viable but non-cultivable (VBNC), which renders it unable to be detected using many traditional microbiological techniques (Tholozan et al., 1999; Ziprin et al., 2003; Castro et al. 2018). Metabolically driven strain to strain variation in the expression of virulence factors makes targeting these factors problematic for the detection of *Campylobacter* using a single methodology becomes problematic (Hofreiter et al., 2008). While it remains unclear as to how the strain-specific diversity of *Campylobacter* impacts the growth and recovery of the pathogen on selective media, it is highly likely that bias and changes in sensitivity and specificity are fluid and environmentally driven through unknown biological interactions.

Consequently, there are significant challenges in culturing *Campylobacter* on selective and/or differential media that arise in the presence of other microorganisms, which can likely influence the metabolism of *Campylobacter*. Also, the diversity of poultry-specific matrices may also induce biochemical changes to *Campylobacter*, which further obscure isolation and identification. In order to assess whether or not microbiologically diverse and complex matrices influence the identification of *Campylobacter*, Oakley et al. (2012) compared five different commercialized selective *Campylobacter* media for the ability to isolate *Campylobacter* from broiler fecal samples. Oakley and colleagues then compared the sequenced colonies from selective media to the pooled fecal samples using 16S rRNA tagged-pyrosequencing. Sequencing results indicated that 0.04% of the total fecal microbial community was *Campylobacter.* Comparing *Campylobacter*-specific media to the sequencing results of the individual colonies indicated that 88–97% of the putative colonies were in fact *Campylobacter* (Oakley et al., 2012). Therefore, when taken together, the specificity of *Campylobacter*-selective media when isolating the pathogen from complex matrices necessitates doubt. Additional data from other studies revealed that specific isolation procedures and culture media influence the diversity of *Campylobacter* species recovered from samples. For example, Ugarte-Ruiz et al., (2013) used eight different isolation protocols, with or without enrichment, followed by culture on selective media. Data indicated that the isolation method used by researchers influenced *Campylobacter* species isolated from poultry samples (Ugarte-Ruiz et al., 2013). Temperature, media, time, and enrichment all influence the ability to isolate *Campylobacter.*

Perhaps, the most significant data that lend themselves to further doubting the use of selective and/or differential media for the isolation of *Campylobacter* emerged with data from next-generation sequencing studies. In a more recent study, Kim et al. (2017a) compared poultry carcass rinsate directly with microbiome sequencing and then to the sequences of the pooled colonies recovered from commercial *Campylobacter* on selective media. Despite the results of selective media, the *Campylobacter* was not a predominant bacterium identified in the rinsate microbiota despite dominating the *Campylobacter* selective plates. While initially not surprising, at different stages of poultry processing, there were a significant range of non-*Campylobacter* bacteria that cocultured on selective media. Common cocultured genera include *Oscillospira, Acinetobacter, Enterococcus,* and *Bacillus* (Kim et al., 2017a). Therefore, data indicate that environmental influences, such as those found at different stages of poultry processing, drive the challenges associated with culturing of *Campylobacter* on selective media. As a result, the traditional culture-based microbiological techniques for the detection of *Campylobacter* in poultry processing must be scrutinized and alternative methods need development. The following sections will focus on the available molecular methods that enable more specific and sensitive detection and quantitation of *Campylobacter* spp. from food and poultry samples.

## Rapid Detection of *Campylobacter*—General Concepts

Over the past few decades, the rapid, culture-independent detection of bacterial pathogens has become increasingly routine. For the detection of foodborne pathogens, there are two classes of technologies used for the fast identification of pathogens: immune and nucleic acid-based methodologies. The immune-based detection methodologies exploit the affinity of antibodies for specific target antigens found on the surface of the desired microorganism. The use of nucleic acid-based technologies recognizes unique and highly specific DNA or RNA sequences that can either be sequenced, amplified and visualized on a gel, or otherwise differentiated for detection, quantification, and molecular typing (Yolken, 1988; Manfreda and De Cesare, 2005; Maciorowski et al., 2006; Mandal et al., 2011; Gharst et al., 2013; Park et al., 2014; Välimaa et al., 2015; Baker et al., 2016; Chen and Park, 2016; Zeng et al., 2016). Both immune and nucleic acid-based approaches are summarized in Table 1 (Immune) and Table 2 (Nucleic-acid). Because these methods can be quick and accurate, kits are actively being commercialized to detect foodborne pathogens. Also, once commercialized, immune and nucleic acid-based assays have the potential to become high throughput. High-throughput assays that are simple to use with a fast turn-around time are imperative for any technology to truly be successful in displacing common microbiological techniques used in poultry monitoring systems. Additionally, having a significant level of repeatability and reliability will also become important to satisfy regulatory bodies that monitor poultry productions. While these approaches have their weaknesses, namely time, specificity, and the limits of detection and quantification, it likely is a necessary step to modernize the food industry. By modernizing monitoring strategies industry-wide, novel insight may fill knowledge gaps associated with the transmission of foodborne diseases (Hannson et al., 2016). Finally, by creating a faster monitoring regime, real-time management decisions that are economical while safeguarding the food supply are possible (Hannson et al., 2016).

**Table 1:** Detection of *Campylobacter* spp. using various immunological techniques.

Target epitope	Organism	Type	Author; Notes
**Monoclonal assays**			
Flagellin	*Campylobacter* spp.	Monoclonal antibodies	Nachamkin and Hart (1986)
Outer membrane protein	*Campylobacter* spp.	Monoclonal antibodies	Lamoureux et al. (1997)
15-kDa cell surface protein	*C. jejuni/C. coli*	Monoclonal antibodies	Kawatsu et al. (2008)
Two *C. jejuni*/one *C. coli* epitope	*C. jejuni/C. coli*	Monoclonal antibody 33D2	Heo et al. (2009)
Lipopolysaccharide antigens	*C. jejuni*	Monoclonal antibodies	Brooks et al. (1998)
Hippurate hydrolase	*C. jejuni*	Monoclonal antibodies	Steele et al. (2002)
Outer membrane protein	*C. jejuni*	Monoclonal antibodies	Qian et al. (2008)
**Nonconventional assays**			
Surface antigen	*Campylobacter* spp.	Enzyme-linked fluorescent assay	Reis et al. (2018); VIDAS® 30 system
Surface antigen	*C. jejuni/C. coli*	Microplate EIA assay	Granato et al. (2010); Premier CAMPY EIA kit
Surface antigen	*C. jejuni/C. coli*	Microplate EIA assay	Granato et al. (2010); ProSpectT Campylobacter EIA kit
Surface antigen	*C. jejuni/C. coli*	Lateral-flow EIA assay	Granato et al. (2010); ImmunoCARD STAT! CAMPY kit
Surface antigen	*C. jejuni*	Cotton swab colorimetric assay	Alamer et al. (2018); swabs contain colored nanobeads with monoclonal antibody cocktails
Surface antigen	*C. jejuni*	Biosensor	Masdor et al. (2016); biosensor with sandwich assays
Surface antigen	*C. jejuni*	Single-chain variable fragment antibodies	Nzuma et al. (2018); detection with IMS-qPCR

**Table 2:** Advantages and disadvantages of selected detection methods.

	Advantages	Disadvantages
**Conventional techniques**		
Selective plating	Inexpensive	Cannot culture VBNC state cells
	Well-established	Variable specificity
	Can customize antibiotic makeup	Can be affected by culturing methods
**Immune-based**		
ELISA	Can perform many samples at once	Loss of sensitivity and specificity in mixed cultures
	Several different possible techniques (direct, indirect, sandwich)	Cross-reactivity between closely related species
	Can change selectivity based on targeted epitopes	False positives from complex matrices
Flow cytometry	Multiple parameters analyzed	Expensive, specialized equipment
	Single cell analysis	Requires highly trained personnel to prepare, run, and analyze data
	High specificity	Relatively slow
**PCR-based**		
Conventional PCR	Better specificity than plating	Non-specific binding of similar DNA
	Can be combined with other assays such as ELISA	Must be optimized
	Relatively simple and quick	Can only be used for presence/absence
Multiplex PCR	Assay multiple species at once	Requires highly specific primers
	Higher throughput than conventional PCR	Difficult to optimize
	Less costly than running multiple assays	False negatives/positives
qPCR	High sensitivity	Complex matrices may include inhibitors
	Used for rapid detection	Require highly specific primers
	May be multiplexed	Cannot differentiate live/dead cells
	EMA/PMA may be used to help distinguish dead cells	
dPCR	Cheaper than qPCR	Cannot differentiate live/dead cells
	Less vulnerable to inhibitors than qPCR	Greater chance of false positives than qPCR
	No calibration or internal controls required	
**Sequencing**		
16S rRNA	Highly conserved region found in all bacteria	High cost of equipment
	Able to distinguish species using variable regions	Relative abundance may be skewed by copy number
	Can be used on non-culturable bacteria	Possible species level resolution issues
Whole genome sequencing	Open access of many databases	High cost of equipment
	High discrimination	Specialized training required
	Can detect antimicrobial resistances and virulence genes	Varied interpretation of data

As a society, we are at the precipice of significant changes arising from the innovations associated with next-generation sequencing and proteomic technologies. Such innovations not only impact molecular genetics and biology but can also be used to improve immune-based technologies. These changes will definitively improve the monitoring of food supplies for pathogens, as well as a host of other opportunities for the poultry industry. Importantly, as the genomes of particular species continue to be identified, the knowledge of foodborne pathogen biology and plasticity will co-evolve. This may be particularly important for an organism such as *Campylobacter,* which can exist as VBNC. Being able to understand specific environmental cues for triggering the VBNC status of *Campylobacter* may help researchers understand its survival patterns and metabolic dependencies. Said knowledge is exploitable as it will naturally lead to improved detection techniques targeted to detect VBNC *Campylobacter*. However, there are also drawbacks to this approach (Table 3). Currently, drawbacks include assay cost and the requirement for higher-skilled workers, and the requirement to update present and future technologies may be problematic. The following sections will describe the current rapid detection and identification methods for foodborne *Campylobacter* in poultry.

**Table 3:** Recent studies in whole genome sequencing (WGS) of *Campylobacter* spp.

Sequencing type	Organism	Topic	Sample; Country; Author; Notes
Illumina	*C. jejuni/coli*	Antimicrobial resistance profiling	Clinical, meats, ceca isolates; USA; Zhao et al. (2016)
			Identified antimicrobial resistance genes to predict phenotypic resistance.
Illumina	*C. jejuni/coli*	Antimicrobial resistance profiling	Poultry isolates; USA; Whitehouse et al. (2018)
			Identified antimicrobial resistance genes to predict phenotypic resistance.
Illumina	*C. jejuni*	Antimicrobial resistance profiling	Clinical, poultry isolates; Estonia; Mäesaar et al. (2018)
			Used WGS and MLST to analyze antimicrobial resistance in strain types.
Illumina	*C. jejuni*	Antimicrobial resistance profiling	Poultry isolates; Europe; Leekitcharoenphon et al. (2018)
			Examined fluoroquinolone resistance in poultry isolates from 12 European countries.
Illumina	*Campylobacter* spp.	Comparative analysis	Poultry, bird isolates; USA; Lawton et al. (2018)
			Compared using MALDI-TOF MS as a rapid method to identify *Campylobacter* isolates to species level.
Ion torrent	*C. jejuni*	Comparative analysis	Clinical, animal, environmental isolates; France; Thépault et al. (2018)
			Studied pathogen source attribution of campylobacteriosis in France using WGS and MLST.
Illumina	*C. jejuni/coli*	Epidemiology	Chicken liver pâté; Sweden; Lahti et al. (2017)
			Outbreak of campylobacteriosis due to undercooked chicken liver pate.
Illumina/wgMLST	*C. jejuni*	Epidemiology	Clinical, poultry, bovine isolates; Israel; Rokney et al. (2018)
			Used WGS and wgMLST to screen for virulence genes in Isreaeli *C. jejuni* isolates.
Illumina	*C. jejuni*	Genome sequence	Poultry isolates; USA; Taveirne et al. (2017)
			Sequenced three *C. jejuni* strains from naturally colonized farm-raised chickens.
Illumina/SMRT	*C. jejuni*	Genome sequence	Poultry isolates; USA; Sacher et al. (2018)
			Sequenced three phage-propagating strains of *C. jejuni*.
Illumina/SMRT	*C. coli*	Genome sequence	Poultry isolate; USA; Ghatak et al. (2017)
			Discovered type VI secretion system and antimicrobial resistance genes in plasmid of *C. coli* YH502.
Ion Torrent	*C. jejuni/coli*	Genome sequence	Chicken sushi; Japan; Asakura et al. (2017)
			Sequenced two isolates associated with an outbreak due to consumption of undercooked chicken sushi.
Illumina	*C. jejuni*	Genome wide association study	Clinical, animal, environmental isolates; Canada; Buchanan et al. (2017)
			Identified gene markers associated with clinically related *C. jejuni* isolates.
Illumina	*C. jejuni/coli*	Stress resistance	Clinical, poultry isolates; UK; O’Kane and Connerton (2017)
			Analyzed aerotolerant *C. coli* strain.

## Immune-Based Assays for *Campylobacter*

The immune-based methodology for the detection of foodborne pathogens has been well established and includes enzyme-linked immunosorbent assays (ELISA), flow cytometry, and quantitative immunofluorescence, among others (Yolken, 1982, 1988; Maciorowski et al., 2006; Bordeaux et al., 2010; Oyarzabal and Battie, 2012; Baker et al., 2016; Alahi and Mukhopadhyay, 2017). Both monoclonal and polyclonal antibodies can be manufactured specifically to detect pathogen-specific epitopes. Additionally, antibodies can be modified, which commonly includes the conjugation of various detection systems, such as horseradish peroxidase, to improve the detection sensitivity and specificity of various target epitopes (Yolken, 1982; Preiner et al., 2014; Janda et al., 2016; Alahi and Mukhopadhyay, 2017).

Immunological methods dedicated to the detection and quantification of *Campylobacter* spp. have been extensively reviewed by Oyarzabal and Battie (2012) and will only be briefly discussed in the current review. Much of the early research focused on the discovery of conserved, non-species-specific *Campylobacter* antigens targeted by monoclonal antibodies, such as lipopolysaccharides, flagellin, and other protein antigens for detection immunoassays (Nachamkin and Hart, 1986; Lamoureux et al., 1997; Brooks et al., 1998; Kawatsu et al., 2008). An example of this was the work conducted by Nachamkin and Hart (1986) where researchers generated murine monoclonal antibodies to one of two distinct *Campylobacter* flagellin epitopes, and in turn became capable of detecting *Campylobacter* spp. and also differentiate *C. jejuni* or *coli*. However, as with most antibody-based biotechnology, there is significant cross-reactivity with *C. jejuni* and *C. coli* as their genetic divergence is not significant (Table 1). Other methodologies include those developed by Steele et al. (2002). Steele and colleagues used an ELISA to screen 11 monoclonal antibodies against *C. jejuni* hippurate hydrolase, or hipO, an enzyme specific to *C. jejuni*. They were able to identify several antibodies that demonstrated a high affinity for *C. jejuni* when exposed to cell extracts from *Campylobacter* and non*-Campylobacter* bacterial species. Research continued to evolve to reduce the cross-reactivity as time progressed. Qian et al. (2008) focused on a major outer membrane protein epitope that specifically targeted *C. jejuni*. Importantly, once researchers isolated the high-affinity *C. jejuni* antibody, they located the epitope by mapping it to a 13-amino-acid polypeptide and confirmed it by demonstrating that alteration of any of the amino acids contained within that epitope reduces antibody binding affinity. Further advancements also included the ability to generate monoclonal antibodies that bind to extremely conserved, linear epitopes not impacted by heat-killing *Campylobacter*, thus enabling the detection of the species in potentially thermophilic environments like a scalder (Heo et al., 2009).

Since the development of immunological-based methods to detect *Campylobacter,* several commercial immunoassays have come on the market and have been compared with non-immunological detection methods (Table 1). Again, such steps are necessary as the continued improvement of technology lends itself to more sensitive and specific assays, which then require validation for the poultry industry. A classic example of the validation of commercial kits that demonstrated superior performance as compared to conventional methods was described by Granato et al. (2010). While the kits were specific for clinical diagnostics, the methodology of comparing the gold standards (qPCR and microbiology) versus newer technology (EIA) is demonstrative of the rigor required for the validation of kits in the poultry industry. Granato and colleagues compared conventional *Campylobacter* microbiological culturing techniques with three commercial *Campylobacter* enzyme immunoassay (EIA) kits as well as a real-time polymerase chain reaction (qPCR). Remarkably, all three EIA commercial kits demonstrated sensitivity and specificity above 98% as compared to 94.1% for the culture methods (Granato et al., 2010). Necessarily, if employing these methods in the poultry industry is the goal, similar metrics must be met to demonstrate that faster methods do not sacrifice sensitivity and specificity. Additionally, as previously mentioned, repeatability is important. In the case of EIA assays, Regnath and Ignatius (2014) also observed a similar agreement to Granato and colleagues when comparing EIA and culture methods for the detection of *C. jejuni* and *coli* from stool samples.

However, there are significant limitations associated with immune-based detection methods. Further comparisons of commercial kits with traditional microbiological and molecular techniques have suggested that despite advancements, cross-reactivity may occur with *Campylobacter* species leading to false positives. These species are typically not the commonly assayed *C. jejuni* and *C. coli*. As a result, some commercial immunoassays may elicit false positives and undefined variability for clinical samples (Myers et al. 2011; Floch et al., 2012; Couturier et al., 2013; Gharst et al., 2013). This lack of sensitivity may result in some evidence suggesting that for poultry samples, qPCR assays may be more sensitive than a commercial enzyme-linked fluorescent assay when tested on chilled and frozen broiler carcasses (Reis et al., 2018).

Challenges associated with matrix-induced loss of sensitivity and specificity have led to the innovation of the technology leading to self-contained immune-based biosensors and nano-based assays. These biosensors convert the binding activities of the antibody into an electrical signal that is more precise for the assessment of the pathogen in a mixed culture (O’Connell et al., 2000; Willner, 2002; Reshetilov, 2005; Wei et al., 2007; Huang et al., 2010; Masdor et al., 2016; Alahi and Mukhopadhyay, 2017; Asal et al., 2018). In a more recent study, Masdor et al. (2017) covalently attached rabbit polyclonal antibodies to gold chips and developed a surface plasma resonance (SPR) sensor platform for *C. jejuni* detection. When the sensor chips were assessed for the limit of detection (LOD), a sandwich format using a polyclonal antibody improved LOD compared to the direct assay. Nano-based immunoassays are potentially practical, user friendly detection strategies for the poultry industry, which can actively be adapted to various poultry-specific applications. For example, Alamer et al. (2018) developed a cotton swab immunoassay for potential use in poultry processing plants by immersing swabs into different colored nanobead-conjugated *Campylobacter jejuni*-specific monoclonal antibody cocktails. The change in color intensity of the swab was captured by a smartphone and quantitated with the National Institute of Health ImageJ computer program. They were able to achieve a LOD of 10 CFU per mL with no observable cross-reactivity. The concept of a cotton swab-based pathogen colorimetric assay would be attractive for assessing contamination in remote sites of poultry processing plants and offer immediate results for making in-plant decisions regarding control measures.

Another potential direction for the improvement of immunoassays would be the use of proteomics to identify ideal target epitopes for monoclonal antibodies to enhance the resolution of differentiating between different strains and species epithets of *Campylobacter.* As *Campylobacter* is genomically and metabolically fluid, this is extremely important for the next generation of EIA development. Rodrigues et al. (2016) used proteomics to demonstrate that there were differences in the cadre of proteins expressed between microaerophilic and aerobic culture conditions. Along these lines, Turonova et al. (2017) uncovered differences in protein expression when *C. jejuni* was switched from the stationary to the exponential growth phase. The application of proteomics to immune responses may prove to be especially helpful to find novel antibodies. Mehla and Ramana (2017) employed computational analyses based on informatics databases of the *Campylobacter* genome to identify and predict immunogenic epitopes that could stimulate B- and T-cell activity and serve as antigens for vaccine construction. It is not difficult to imagine that similar data mining approaches could be used to identify antigens for specific monoclonal antibody generation and EIA development that would enable optimal sensitivity and specificity. While easier said than done, data could then be used to develop antibodies that are robust to matrix inhibition and target highly specific and conserved epitopes.

Advances in proteomics-based antibody production technologies will improve both sensitivity and specificity as well as provide alternative sources for hybridoma-based antibody production. Alternative antibody production systems include development of alternative antibody sources such as cloning antibody variants into yeast or bacterial cultures, and perhaps even creating polyclonal antibodies in transgenic plant expression systems (Berghman et al., 2005; Baker et al., 2016). New opportunities exist that will be revolutionary for the poultry industry. For example, Nzuma et al. (2018) recently constructed recombinant single-chain fragment variable (scFv) *C. jejuni* antibodies with a scFv antibody phage-display library and spleen mRNA from *C. jejuni* immunized rabbits and subsequently purified the antibodies. The resulting purified *C. jejuni* scFv antibody was covalently bound to paramagnetic beads for use in an immunomagnetic separation (IMS) capture system, which was ran in parallel with qPCR, for the detection of *C. jejuni* successfully. The continued evolution of this technology may even allow for the real-time monitoring of poultry processing, which would be a momentous achievement. In the future, achieving a more precise identification of suitable epitopes with advanced bioinformatics makes it even more conceivable that immunoassay improvement will dramatically eliminate most of the current shortcomings associated with current assays.

## Conventional PCR and *Campylobacter*

Molecular methods for identifying and detecting foodborne pathogens have become more sensitive as comprehensive genomic data continue to be generated from foodborne pathogens (Gharst et al., 2013; Park et al., 2014; Baker et al., 2016). One of the most valuable techniques to be employed by the food safety industry, and science as a whole, is polymerase chain reaction (PCR). The use of PCR is the foundation for numerous technologies. At its core, PCR uses specific oligonucleotides to bind to either unique genes carried by individual species or a single sequence that exhibits sequential or length variation and amplifies it. Therefore, PCR exploits the genomic nuances of a particular organism to differentiate it from unrelated and related organisms (Eisenstein, 1990; Carrino and Lee, 1995; Hill, 1996). A selected group of PCR primers, their gene targets, and experimental samples is presented in Table 4.

**Table 4:** Gene targets for the detection of *Campylobacter* spp. using various polymerase chain reaction (PCR) techniques.

Target gene	Organism	Primers	Sample; Country; Author; Notes
**Traditional and multiplex PCR**			
16S rRNA	*Campylobacter* spp.	3’-ACCTTGTTACGACTTCACCCCA-5’5’-GAGAGTTTGATCCTGGCTCAG-3’	Chicken wings; Netherlands; Giesendorf et al. (1992)
16S rRNA	*Campylobacter* spp.	5’-AATTCTAATACGACTCACTATAGGGAGAGTGTGACTGATCATCCTCTCA-3’5’-GACAACAGTTGGAAACGACTGCTAATA-3’	Poultry products, dairy products, red meat, vegetables; Belgium; Uyttendaele et al. (1995)
16S rRNA	*Campylobacter* spp.	5’-GGATGACACTTTTCGGAGC-3’5’-CATTGTAGCACGTGTGTC-3’	Fecal samples; Australia; Linton et al. (1996), Huq et al. (2014)
256bp fragment	*Campylobacter* spp.	5’-AGAACACGCGGACCTATATA-3’5’-CGATGCATCCAGGTAATGTAT-3’	Poultry, beef, pork samples; USA; Jackson et al. (1996a,b), Zhao et al. (2001)
			Multiplex capable, see text for details
Flagellin -	*C. jejuni/coli*	5’-ATGGGATTTCGTATTAAC-3’	Fecal samples; USA; Oyofo et al. (1992)
*flaA*		5’-GAACTTGAACCGATTTG-3’	
Flagellin -	*C. jejuni/coli*	5’-CCAAATCGGTTCAAGTTCAAATCAAAC-3’	Fecal samples; Denmark; Guerry et al. (1990), Rasmussen et al. (1996)
*flaA, flaB*		5’-CCACTACCTACTGAAAATCCCGAACC-3’	
Heat shock protein -	*C. jejuni*	5’-CAAGTTGCTACAATCTCAGCCA-3’	Water samples; USA; Park et al. (2011)
*hsp60*		5’-GATAACACCATCTTTGCCCACT-3’	Multiplex capable, see text for details
Hippuricase -	*C. jejuni*	5’-GACTTCGTGCAGATATGGATGCTT-3’	Fecal samples; Denmark; Persson and Olsen (2005)
*hipO*		5’-GCTATAACTATCCGAAGAAGCCATCA-3’	Multiplex capable, see text for details
Cytolethal distending toxin -	*C. jejuni*	5’-AGGACTTGAACCTACTTTTC-3’	Broiler carcasses, vegetable samples; Brazil; Asakura et al. (2007), Carvalho et al. (2013)
*cdt*		5’-AGGTGGAGTAGTTAAAAACC-3’	Multiplex capable, see text for details
Aspartokinase -	*C. coli*	5’-GGTATGATTTCTACAAAGCGAG-3’	Fecal samples; Denmark; Linton et al. (1997), Persson and Olsen (2005)
*asp*		5’-ATAAAAGACTATCGTCGCGTG-3’	Multiplex capable, see text for details
Lipid A acyltransferase -	*C. upsaliensis*	5’-CGATGATGTGCAAATTGAAGC-3’	Biochemical assays; Japan; Yamazaki-Matsune et al. (2007)
*lpxA*		5’-TTCTAGCCCCTTGCTTGATG-3’	Multiplex capable, see text for details
**qPCR and dPCR**			
16S rRNA	*Campylobacter* spp.	5’-CTGCTTAACACAAGTTGAGTAGG-3’5’-TTCCTTAGGTACCGTCAGAA3’	Chicken carcass rinses; Denmark; Josefsen et al. (2004), Josefsen et al. (2010); **PMA-PCR**
Fragment of *C. jejuni*	*C. jejuni*	5’-CTGAATTTGATACCTTAAGTGCAGC-3’5’-AGGCACGCCTAAACCTATAGCT-3’	Viable/dead cells; Norway; Nogva et al. (2000), Rudi et al. (2005); **EMA-PCR**
16s rRNA	*Campylobacter* spp.	5’-GGATGACACTTTTCGGAGC-3’5’-CATTGTAGCACGTGTGTC-3’	Fecal samples; UK; Logan et al. (2001); **qPCR**
ATP binding protein -	*C. jejuni/coli*	5’-AGTGCCGATAAAGGCTCATCA-3’	Poultry, fish, beef, pork, milk, vegetable samples; Spain; Bonjoch et al. (2009); **qPCR**
*cje0832*		5’-ACTCGTCGAGCTTGAAGAATACG-3’	
VS1 gene	*C. jejuni*	5’‐GAATGAAATTTTAGAATGGGG‐3’5’‐GATATGTATGATTTTATCCTGC‐3’	Chicken, milk, water; China; Yang et al. (2006); **qPCR**
Hippuricase -	*C. jejuni*	5’-TCCAAAATCCTCACTTGCCATT-3’	Poultry processing water; USA; He et al. (2010), Rothrock Jr et al. (2013); **ddPCR**
*hipO*		5’-TGCACCAGTGACTATGAATAACGA-3’	
Cytochrome c oxidase -	*C. jejuni*	5’-TGGTCTAAGTCTTGAAAAAGTGGCA-3’	Broiler neck-skin; Slovenia; Toplak et al. (2012), Papic et al. (2017); **ddPCR**
*ccoN*		5’-ACTCTTATAGCTTTTCAAATGGCATATCC-3’	

Numerous PCR-based approaches have been successful for the detection of *Campylobacter* species commonly found in poultry. While significant epidemiological evidence suggests that there are mainly two clinically important isolates of *Campylobacter*, the more advancements achieved in genomics and molecular biology, the more it will become necessary to differentiate between non-classical isolates of *Campylobacter*. A central target for differential PCR assays includes the 16S rDNA gene. Early efforts by Giesendorf et al. (1992) focused on PCR primers that targeted the variable regions of the 16S rRNA genes of three *Campylobacter* species: *C. jejuni*, *coli*, and *lari*. By doing so, researchers enabled a lower detection limit to approximately 12 colony forming units (CFU) as compared to traditional methods. In order to further distinguish between non-*C. jejuni* and *coli* species, Linton et al. (1996) successfully identified 16S rRNA sequences specific for five different *Campylobacter* species, namely: *C. upsaliensis*, *helveticus*, *fetus*, *hyointestinalis*, and *lari.* Researchers targeted PCR oligonucleotides for each species epithet along with a genus-specific *Campylobacter* oligonucleotide, which were created and validated. Taking a different approach, Uyttendaele et al. (1995) also targeted 16S rRNA to detect *C. jejuni* by combining selective enrichment and nucleic acid sequence-based amplification (NASBA) of RNA to shorten the assay time of food samples from 6 days to 26 h.

Other gene targets have also been used over the years, which exploit the carriage of gene-specific differences in closely related *Campylobacter* species. Oyofo et al. (1992) developed PCR assays based on the species-specific upstream regions of the flagellin genes *flaA* and *flaB* to differentiate *C. jejuni* and *C. coli. Campylobacter* spp. and non-*Campylobacter* genera were included in the analysis to evaluate the specificity of detection. Using a purified template, the assay achieved an overall detection sensitivity of 98.5%, which included the discrimination between *C. jejuni* and *C. coli* despite the similarities in flagellin gene sequences. Studies such as those conducted by Oyofo and colleagues are important as mixed poultry matrices include other, perhaps undefined, *Campylobacter* species as well as members of the family *Enterobacteriaceae* that share the same gene. Therefore, targeting an upstream region of the *flaA* and *flaB* genes to successfully identify and differentiate *C. jejuni* and *C. coli* is crucially important for the poultry industry. As a result, multiple labs continued to target the *flaA* and *flaB* genes, including Rasmussen et al. (1996), who created oligonucleotide probes hybridized to a microtiter plate targeting both flagellin genes to improve sensitivity (less than two cells for *C. jejuni*) and specificity of the detection of *Campylobacter* in chicken fecal samples.

Even though PCR is an improvement as compared to traditional microbiology-based culture methods, the successful and specific detection of *Campylobacter* in mixed matrices continues to be a challenge. This variability is influenced by several factors, such as the presence of non-culturable bacteria (Leskinen and Lim, 2008), polymerase inhibitors, fecal material (Loge et al., 2002), and low quantities of cells existing in a large volume of sample. Innovations to PCR-based techniques have addressed many of those limitations, such as reducing inhibitors, including an enrichment step before the PCR, and coupling the PCR assay with other assays like EIA to enhance assay sensitivity (Park et al., 2014). For example, by combining PCR with ELISA, researchers detected *Campylobacter* in environmental water samples that were below the limit of detection of conventional cultural methods (Sails et al., 2002).

On et al. (2013) emphasized that as new *Campylobacter* spp. are discovered, it is critical to revalidate existing PCR assays for *C. jejuni* and *C. coli* to reconfirm species specificity and avoid false positive results. Multiple clinical laboratories evaluated 31 different *Campylobacter* PCR assays to detect and differentiate *Campylobacter* species. The overall results for sensitivity (number of true positives/true + false positives) ranged from 0 to 100% and specificity (number of true negatives/true negatives + false positives) ranged from 55 to 100%. The authors concluded with the recommendation that as the taxon numbers for *Campylobacter* increase, it is critical for diagnostic laboratories dependent on PCR analyses to stay in touch with changes in taxon designations and to obtain the type of strains of new taxa to validate against in-house PCR methods. Campylobacter are extremely fluid organisms and require continuous innovation for their detection. Additionally, it will become increasingly important for testing to become integrative between clinical, epidemiological, and veterinary medicine in order to be all inclusive.

## Multiplex PCR Detection of *Campylobacter*

Multiplex PCR is a quick and reliable method for determining the presence or absence of multiple gene targets within a single sample. This approach has been established for use in *Campylobacter* for the rapid identification of several *Campylobacter* species epithets as well as *Campylobacter* and non-*Campylobacter* efficiently. Therefore, it is an attractive methodology for prevalence testing in the poultry industry as one sample can yield multiple identifications quickly and efficiently.

Multiplex PCR assays for the detection of *Campylobacter* spp. have proven useful for the identification of multiple species in food production samples. Zhao et al. (2001) developed a multiplex PCR to differentiate *Campylobacter jejuni* and *C. coli* and to confirm presumptive *Campylobacter* isolates on blood agar plates. The isolates recovered from the blood agar plates were from retail chicken, turkey, pork, and beef products. While most colonies were confirmed as being either *C. jejuni* (53.6%) or *C. coli* (41.3%), the remaining colonies apparently were other *Campylobacter* spp. not identified by the species-specific multiplex PCR. As several of the meat samples yielded more than one *Campylobacter* species, researchers concluded that because of the likelihood of mixed species contamination, it is critical to select more than one colony per plate for *Campylobacter* identification and subtyping. Data from the study also emphasized the need for expanding the multiplex PCR design to cover more *Campylobacter* species and to develop culture-independent methods.

Despite the importance of purely culture-independent methods being developed, typically, culturing *Campylobacter* may be a crucial first step to its identification in complex samples. Multiplex assays have the ability to very specifically determine if *Campylobacter* is present. The importance of being able to identify multiple *Campylobacter* species directly from single, yet diverse samples is supported by Inglis and Kalischuk (2003) when they compared four different *Campylobacter* media with direct PCR detection without the pre-enrichment of bovine fecal samples. The genus level identification for *Campylobacter* was generally more sensitive (8% improvement) as compared to a broad survey of microbiological isolation techniques that included four different media and three different incubation temperatures. While the detection of *C. lanienae* by PCR was more sensitive than microbiological isolation, this was not the case for *C. jejuni.* The authors suggested that this variability in isolation prevalence of *Campylobacter* spp. on selective media could be due to an inherent bias of selective media. Specifically, researchers theorized that in the case of *Campylobacter*, the media used may specifically enhance the growth and isolation of *C. jejuni* and *C. coli* and select against the less defined species epithets that may be present.

In another study, Huq et al. (2014) also saw an improved sensitivity using a multiplex PCR to identify *C. concisus*, *C. jejuni*, and *C. coli* in spiked human fecal samples from clinical gastroenteritis cases as compared to traditional microbiological techniques utilizing an antibiotic-free Columbia blood agar using the micron filtration technique. However, evidence suggests that depending on the sample, using selective media prior to PCR can improve sensitivity. For example, Persson and Olsen (2005) demonstrated that human stool samples spiked with *C. jejuni* and *C. coli* that were plated on modified charcoal cefoperazone deoxycholate agar (mCCDA) improved the sensitivity of the PCR assay to 10^2^ cells per ml of stool from 10^5^ cells per ml of stool with direct multiplex PCR detection alone. In short, comparisons in sensitivity between direct PCR and culture methods versus selective enrichment-PCR combinations are likely a result of viability status of *Campylobacter* spp. in different samples and the caveats therein. Meaning, various samples contain different inhibitors present in the sample and can augment the sensitivity and specificity of the assay. Additionally, enrichment may overcome cell counts below the limit of detection, which may be important for monitoring poultry in the pre-harvest or peri-harvest setting. As a result, developing standard protocols will likely require independent validation for the type of sample being tested. While many of the aforementioned methodologies are clinically based, the complexity of human stool samples and the importance of highly specific and precise molecular identification of *Campylobacter* are essential.

Unlike immune-based methods that have challenges associated with the identification of closely related species, molecular PCR-based approaches may be able to offer significantly more detailed refinement and precision. Evidence suggests that in poultry, methods may need to go beyond classical genus identification, specifically *C. jejuni* and *C. coli.* The identification of *Campylobacter* may need to include the less characterized species like *C. lari*, *C. upsaliensis*, and *C. fetus* as well as other important foodborne pathogens simultaneously (Wang et al., 2002; Klena et al., 2004). Yamazaki-Matsune et al. (2007) expanded the range of *Campylobacter* by developing a multiplex PCR for six individual species of *Campylobacter*. Researchers designed oligonucleotides targeting *lpxA* in combination with previously published primers (16S rRNA, 23S rRNA, *ask*, *cstA*, *glyA*, and *cj0414*) to differentiate between *C. coli*, *C. fetus*, *C. hyointestinalis* subsp. *hyointestinalis*, *C. jejuni*, *C. lari*, and *C. upsaliensis*. Other laboratories have developed and applied a multiplex PCR for *Campylobacter* spp. using oligonucleotides to target the cytolethal distending toxin (*cdt*) genetic subunits (Carvalho et al. 2013; Kamei et al., 2016). Another example of this methodology that may be important for the poultry industry was conducted by Park et al. (2011). Park and colleagues developed multiplex a PCR assay that could detect several pathogens in a single reaction, including *Campylobacter*, *E. coli*, and *Salmonella* Typhimurium simultaneously. In a later study, Raja et al. (2016) developed a multiplex PCR for simultaneous detection of *Campylobacter jejuni* and *Listeria monocytogenes* in chicken meat.

The sensitivity and specificity of PCR, as well as the detection capabilities of the technology, are further improved with the use of nested PCR. Nested PCR starts with a single, long amplicon containing all of the genes of interest, which is then amplified in a second reaction. This overcomes difficulties like secondary structure and specificity challenges associated with two very closely related species or sub-species. This technique can be combined with a multiplex assay to differentiate sub-species of *Campylobacter*. Miller et al. (2007) further improved the genomic resolution by designing a nested multiplex assay with oligonucleotides targeting the nitrate reductase (*nap*) locus with flanking *napA* and *napB* primer sets for simultaneously distinguishing the two *C. jejuni* species: subsp. *jejuni* and subsp. *doylei*. Specific nuances between the two subspecies of *Campylobacter* resided in nitrate metabolism, where subspecies *doylei* is unable to reduce nitrate. Further, based on DNA microarray analyses, it was validated that *napA* and *napB* were either missing or their sequences were unique as compared to the subspecies *jejuni napA* and *napB*. Consequently, subsp. *doylei* strains failed to amplify with the *napA* internal primer set, but could amplify with the flanking *napA* primers and result in a smaller amplicon due to a deletion within *napA*. Further refinement of this method became possible after sequencing data of the subsp. *doylei napA* and *napB* revealed that while all subsp. *doylei* possessed identical *napA* deletions, some strains also contained *napB* deletions compared to others where *napB* was intact. Primers were then redesigned to exploit that difference and improve the detection of these subspecies.

As with other methods, there are drawbacks to the technology, as delineated in Table 2. Despite the attractiveness of using multiplex PCR to simultaneously detect several *Campylobacter* spp., Park et al. (2014) have pointed out that there are limitations associated with involving more than 5–6 primer pairs in a single reaction with less than 100 bp differences between amplicons. The amplification of amplicons not adhering to those guidelines is difficult using a standard agarose gel electrophoresis. Additional primer sets to the mix also reduce the ability to optimize thermocycling as the T_m_ ranges between the oligonucleotides will likely become more difficult to control. Several approaches have been developed to overcome this issue, from changing the percentage of agarose to adjusting the buffers for electrophoresis. This has resulted in some researchers being able to overcome these limitations. To achieve the simultaneous detection of nine different foodborne pathogens, including *C. jejuni*, by multiplex PCR, Villamizar-Rodríguez et al. (2015) combined the enrichment of microbial populations using a universal pre-enrichment medium followed by PCR amplification with nine different primer pairs, with one unique oligonucleotide pair for each respective foodborne pathogen. To circumvent the difficulties with agarose gel electrophoresis, the authors differentiated the various sizes by capillary electrophoresis. When cultural and multiplex PCR results were compared for various spiked food matrices, coincident values ranged from 78 to 92%.

## Real-Time Quantitative PCR (qPCR) and *Campylobacter*

An evolution in PCR technologies for the absolute quantification of pathogens and nucleic acid sequences became possible once intercalating fluorescent dyes that could be detected with specialized laser-imaging systems were introduced. Fluorescent dyes accumulate with each cycle with an intensity that is directly proportional to the amount of target template DNA. This technology is referred to as real-time PCR or quantitative PCR, which are both collectively known as qPCR (Higuchi et al., 1993; Park et al., 2014; Kralik and Ricchi, 2017). The increased sensitivity of fluorescence detection and the implementation of specialized cameras reduce the time it takes to detect a specific gene segment (Mackay, 2004). Fluorescence can either be introduced to the reaction through non-specific dyes that bind to the DNA, such as SYBR green, or by using probes with different fluorescent labels, which is commonly referred to as the TaqMan system. The use of fluorescent probes or melt temperatures allows for multiplexing based on fluorophore emission spectra (Wittmer et al., 2001). The speed and specificity of qPCR enabled the widespread implementation of its use for rapid detection and quantification of foodborne pathogens.

The technical aspects of qPCR and its application to the food industry have been reviewed recently (Chapela et al., 2015; Kralik and Ricchi, 2017); therefore, the following discussion will focus on the development of qPCR for *Campylobacter* in poultry production systems. Yang et al. (2003) developed a qPCR assay based on *VS1* of *C. jejuni* for quantitation in naturally contaminated poultry, milk, and environmental water samples. Without using culture-based enrichment, researchers achieved a sensitivity of 1 CFU in poultry breast, drumstick, and other poultry samples. This was statistically different from culture-based detection methods. Importantly, all poultry samples that tested positive for *Campylobacter* using qPCR were also positively identified on *Campylobacter* selective medium. However, some of the culture negative samples were qPCR positive, further highlighting the difficulties of traditional culture-based methods for the detection of *Campylobacter* in complex matrices.

As with other methods delineated in this review, a significant challenge exists in the detection of *Campylobacter* in diverse samples like ceca due to inhibitors present in the matrices or other unforeseen biological challenges. One way to overcome this inhibition was executed by Lund et al. (2004), who overcame qPCR inhibition in poultry fecal samples by using magnetic beads to separate the DNA. In order to control the uneven loss of DNA, researchers added a known amount of *Yersinia ruckeri* to the unsorted ceca, which is a fish pathogen not native to chickens, as an internal control for DNA isolation and qPCR amplification. Researchers did not identify significant differences in performance between qPCR and conventional selective enrichment culturing techniques nor a bias in DNA isolation. Similar to Lund and colleagues, Rudi et al. (2004) used paramagnetic beads for both *C. jejuni* isolation and DNA purification from chicken cecal and fecal samples. They achieved detection between 2 and 25 CFU from spiked cecal and fecal contents, and reduced the total assay time to less than 4 h. The ability to identify *Campylobacter* in ceca rapidly is essential for the poultry industry.

Multiplex qPCR has emerged to enable the simultaneous detection of all major poultry-specific *Campylobacter* genera within a single sample. While multiplex qPCR can be challenging, Chapela et al. (2015) pointed out that qPCR is actually fairly amendable to multiplexing as sequence-specific probes can be labeled with different fluorophores. This enables the sample to be assayed with multiple probes within a single reaction well. Even non-specific SYBR green can still differentiate genes by generating distinct melt curves for each gene that can be tracked and analyzed. For example, Park et al. (2011) evaluated water samples using different melting temperatures to quantify *C. jejuni* (80.1°C), *E. coli* O157:H7 (83.3°C), and *Salmonella* Typhimurium (85.9°C) in a single reaction. Both the qPCR and culturing methods indicated a reduction in viable cells in spiked watershed samples after 7 days of cold storage (4°C). In a later study, Barletta et al. (2013) successfully took a similar approach to differentiate *Campylobacter*, *Salmonella*, and *Shigella* with a SYBR green-based multiplex qPCR analysis of spiked stool samples.

As with conventional PCR, the ability to differentiate between various species of *Campylobacter* using qPCR and multiplexed qPCR is important. To distinguish four different *Campylobacter* spp., Bonjoch et al. (2009) used two regulatory gene primers from the *bipA* gene for detection of *C. upsaliensis* and *C. lari* and the adenosine triphosphate (ATP)-binding protein CJE0832 for *C. coli* and *C. jejuni*. They analyzed a wide range of meat, fruit, and vegetable samples (200 total) including minced chicken, chicken drumsticks, and turkey legs. The samples were screened with both the qPCR assay and a cultural method. No false negative results were detected and 100% of the *Campylobacter* positive samples were confirmed. Likewise, when Reis et al. (2018) compared qPCR *Campylobacter* with an immunoassay and conventional PCR on frozen and chilled broiler carcasses, the qPCR results were more sensitive.

Several refinements have improved the qPCR methods for the detection and quantitation of *Campylobacter*, which are translatable to the poultry industry. As with other techniques, researchers have actively evaluated whether or not combining qPCR with enrichment or quantitation methods improves detection. The specific quantitation of *Campylobacter* relative to CFUs was improved by Banting et al. (2016), which is important as quantitation and prevalence both matter to the poultry industry. Banting and colleagues combined qPCR with a miniaturized most probable number (MPN), which is a standard for bacterial quantitation in the poultry industry. In this study, water samples were added to microtiter plates and were incubated, and then analyzed for *Campylobacter via* qPCR. For *Campylobacter* positive samples, additional qPCR assays were conducted to identify specific *Campylobacter* spp. Based on one of the specific qPCR assays, *Campylobacter* was estimated to be as low as less than 2 MPN per 300 ml and *C. jejuni*, *C. coli*, and *C. lari* were the most frequent isolates. The assay time can be further reduced by using a non-selective medium to increase the growth rate as demonstrated with *Salmonella* (Kim et al., 2017b). A key to these MPN approaches is to lower the limits of detection and to recover injured cells. As the miniaturization of MPN and qPCR combinations continues to evolve, along with incorporation of automation, opportunities will be offered to develop high-throughput assays that can be used by the food industry to process much larger sample volumes efficiently.

A key disadvantage of qPCR and other PCR-based technologies is that they cannot differentiate live versus dead cells as they quantify all nucleic acids present that are able to anneal to the oligonucleotides (Kralik and Ricchi, 2017). In order to overcome this potential issue, Kralok and Ricchi suggest either using a pre-enrichment growth stage to recover viable cells, using RNA, or using florescent dyes that penetrate and exploit the physiology of dead cells. The use of fluorescent dyes is increasingly popular as they can penetrate dead cell walls and degradation by dyes. Not surprisingly, the choice of dyes matters. For example, while Rudi et al. (2005) found a favorable comparison between ethidium monoazide (EMA) and propidium monoazide (PMA), Flekna et al. (2007) determined that EMA reduced the presence of viable genomic DNA in live *Campylobacter jejuni* and *Listeria monocytogenes.* Consistent results for PMA also appear to be variable for *Campylobacter* live-cell detection. Josefsen et al. (2010) compared a PMA-qPCR assay with the isolation of *C. jejuni* from poultry rinsates on modified charcoal cefoperazone deoxycholate (mCCDA) and Abeyta-Hunt-Bark agar plates. Researchers found a correlation between the live-dead qPCR and traditional microbiological plating techniques of 0.844 *R*^2^. This correlation came into question when Pacholwicz et al. (2013) evaluated naturally contaminated carcasses with traditional microbiological enumeration against PMA-qPCR. Researchers did not detect a concordant relationship between the two methods, leading them to suspect that inadequate concentrations of PMA lead to an insufficient repression of dead cell DNA.

As a result, some controversy remains as to which fluorescent dye is the best choice to quantify live populations of *Campylobacter jejuni*. Some studies, such as that of Seinige et al. (2014), observed concordant data with EMA- and PMA-qPCR and traditional microbiological culturing techniques. Krüger et al. (2014) compared EMA with PMA and demonstrated that EMA was insufficiently active in less metabolically active *Campylobacter* cells. Researchers noted that PMA worked across a broad range of *Campylobacter* metabolic states equally, though it was less efficient in inhibiting DNA from dead cells as compared to EMA across the board. Based on this variability, Krüger et al. (2014) suggested incorporating principles of method for quantification, which reflect intact and infectious *Campylobacter*. Given the importance of distinguishing viable versus dead *Campylobacter* in poultry production systems, further refinement of this technique is needed if the methods are going to be incorporated into processing plants.

Other limitations also exist for qPCR, as described in Table 2. Bias in detection and quantitation can occur with various platforms and extraction methods, as well as primer sensitivity and specificity. Caution must be used when extracting DNA from complex organic matrices, such as fecal material and carbohydrate-rich foods, as inhibitors can coprecipitate with the DNA. Therefore, it is important to customize extraction protocols to reduce contaminants that could interfere with PCR amplification (Pontiroli et al., 2011; Park et al., 2014). Methodologies to reduce inhibitory compound contamination include selecting an extraction procedure that minimizes interference or adding in a pre-enrichment step to allow bacteria to grow and then dilute out the inhibitors (Pontiroli et al., 2011; Park et al., 2014; Kralik and Ricchi, 2017). This consideration is true even for commercial DNA extraction kits. For example, when Kawase et al. (2014) compared two commercial fecal DNA extraction kits on human feces to pathogen DNA, they detected higher recovery of target genes and improved detection consistently with one kit versus the other. This was true for both samples spiked with pathogens as well as naturally contaminated samples from outbreak patients.

## Digital PCR (dPCR) and *Campylobacter*

Baker (2012) defined that digital PCR (dPCR) utilizes the fraction of negative replicates to determine the absolute copy number of a gene target as calculated using a Poisson statistical algorithm. This is accomplished by separating a sample into a large number of small reaction chambers. The resulting number of positive versus negative reactions reveals the exact number of copies of a particular gene in the test sample after amplification is complete, unlike qPCR which follows fluorescence intensity during amplification (Baker, 2012). Therefore, dPCR is significantly more precise than qPCR. Another advantage of dPCR over qPCR is that quantification is less vulnerable to enzyme amplification inhibitors present in the sample matrix (Baker, 2012; Huggett et al., 2015; Papic et al., 2017). Variations of dPCR include droplet dPCR (ddPCR), where titrated emulsions of oil, water, and stabilizing chemicals generate droplets that can be placed into tubes in a thermocycler, followed by analysis on a droplet machine (Baker, 2012).

Poultry and *Campylobacter*-based applications for dPCR have been relatively limited thus far. Rothrock et al. (2015) used a ddPCR approach to quantify *Salmonella* spp., *C. jejuni*, and *L. monocytogenes* in water samples from commercial poultry processing facilities. The researchers collected water samples from the scalder and chiller tanks at three time points: prior to carcasses entering the processing plant, midday, and at the end when the last carcasses had been processed. This study was conducted over a three-day period. The same primers and probes were used for both qPCR and ddPCR assays. The data from these assays were compared with those of conventional culture methods for each pathogen. In general, more pathogens were detected at each sampling point by ddPCR than either cultural recoveries or qPCR. In particular, ddPCR detected *C. jejuni* and *L. monocytogenes* in both the scalder and chiller tank water samples throughout the day, even when they were not detected in the culture samples. This could be due to an enhanced sensitivity or due to picking up the residue of dead *Campylobacter.* More recently, Papic et al. (2017) compared qPCR and dPCR for the quantification of *C. jejuni* in broiler neck-skin samples from poultry processing plants in conjunction with the ISO Standard Plate Count Method. There was a statistically average agreement among all three methods. It was also noted that dPCR yielded an overestimate that they attributed to the high number of false positive outcomes. Another explanation could be that with the enhanced sensitivity of detection, the dPCR picks up residues of dead *Campylobacter* post sanitation. Regardless, the potential use of dPCR and ddPCR in the poultry industry is intriguing.

## Genotyping of *Campylobacter*

Accurate methods for identifying and classifying *Campylobacter* isolates that have a short turnaround time are becoming important for rapidly identifying the source of infection, the vehicle for transmission, and the incidence of campylobacteriosis (Dingle et al., 2002). Consequently, genotyping approaches have been implemented for the major foodborne pathogens including *Salmonella*, pathogenic *E. coli*, and *Campylobacter* (Eberle and Kiess, 2012; Gharst et al., 2013; Park et al., 2014; Baker et al., 2016). Early genetic-based identification of *Campylobacte*r spp. focused on applying DNA homology comparisons for classifying and typing *Campylobacter* isolates. For example, Roop et al. (1984) used DNA homology to differentiate 84 strains of catalase-positive *Campylobacter* isolates, classifying them into seven separate DNA homology groups and correlating these groups to biochemical and physiological characteristics.

Recent advances in multiplex qPCR technology have enabled even more sensitive and specific genotyping. Banowary et al. (2015, 2018) employed a high resolution melt (HRM) curve analysis method based on the use of fluorescent DNA binding dyes. This technology differentiates *Campylobacter* PCR product sequence variation and calculates an average HRM genotype confidence percentage. Initial tests with human clinical and chicken swab samples using segments of the hippuricase gene (*hipO*) for *C. jejuni* and the aspartokinase (*asp*) gene for *C. coli* as primer sources allowed for differentiation of the two species in these sample matrices without requiring enrichment (Banowary et al., 2015). The HRM approach may offer more resolution for determining discrimination capability among primers. For example, Banowary et al. (2018) compared two multiplex PCR-HRM methods (mPCR1-HRM and mPCR2-HRM primers from *cadF* and *gpsA* genes, respectively) to detect and distinguish 24 poultry isolates and three reference strains of *C. jejuni* and *C. coli.* They found them to be more discriminatory than the *hipO* and *asp*-based primers used in their previous study (Banowary et al., 2015) and the mPCR1-HRM could differentiate *C. coli* intra-species variation and mPCR2-HRM *C. jejuni* intra-species variation.

Later developments in molecular technologies led to more advanced genotyping methodologies for *Campylobacter* spp. By the early 2000s, several different genotyping approaches had become available for *Campylobacter* including flagellin (*fla*) gene typing, pulsed-field gel electrophoresis (PFGE), ribotyping, random amplified polymorphic DNA (RAPD), AFLP (amplified fragment length polymorphism), multiplex PCR-RFLP analysis, and multi-locus sequencing typing (Wassenaar and Newell, 2000; Eberle and Kiess, 2012). These were described in detail in several previous reviews (Wassenaar and Newell, 2000; Eberle and Kiess, 2012; Taboada et al., 2013) and will not be discussed in the current review.

## Next-Generation Sequencing of *Campylobacter*

Since its inception, next generation sequencing (NGS) methods have rapidly been adopted for both the qualitative assessment of poultry systems and the epidemiological tracking of foodborne outbreaks. This technology consists of whole genome sequencing (WGS) actively being used to elucidate the individual genomes of foodborne pathogens during outbreaks as well as the complex metagenomics of the microbiomes associated with foodborne pathogens (Park et al., 2014; Allard, 2016; Cao et al., 2017; Sekse et al., 2017; Taboada et al., 2017; Ronholm, 2018). Furthermore, the use of benchtop sequencers, like nanopore, only increases the ability of the industry, researchers, and monitoring bodies alike to evaluate the genomics of species in real time (Llarena et al., 2017). The use of WGS has become the preferred diagnostic and surveillance approach for the United States and global food safety regulatory agencies (Taboada et al., 2017).

Certainly, the laboratory advancements in sequence technologies have led to a decrease in costs, and the increased ease of accompanying bioinformatics programs has ushered WGS to the forefront of pathogen monitoring. The increase open access of the data and analytics platforms has led to the rapid dissemination of information available for foodborne pathogens (Sekse et al., 2017; Taboada et al., 2017; Ronholm, 2018). A list of recent research in the whole genome sequencing of *Campylobacter* spp. is presented in Table 3. Several challenges remain, such as the cost, educational burden for data analysis and interpretation, quality, and speed of sequencing (Metzker, 2010; Park et al., 2014; Pennisi, 2016, 2017; Llarena et al., 2017). The NGS databases for foodborne pathogens continue to grow. This improves the data resolution and tracking of outbreaks and makes characterizing taxonomical relatedness within an outbreak more precise. In fact, the use of NGS to pinpoint outbreaks is already common and shows an unusual robustness to the methodology used (Llarena et al., 2017). By focusing on allelic variation and differentiation between isolates, researchers can trace outbreaks with significant discriminatory power (Llarena et al., 2017). The scope and success of WGS approaches can be appreciated in the study of Llarena et al., 2017, with recent advances described in Table 2.

Importantly, the data gained go beyond bacterial identification. Early studies on sequencing of the *Campylobacter* genome revealed the presence of hypervariable regions contained within the loci of genes involved with surface structure biosynthesis or modification (Parkhill et al., 2000). What seems to be unique for the *Campylobacter* genome is that there are very few insertion, phage, or tandem repeat sequences. The usage of these data has been important as it has led to a number of applications for further analyses and characterization of the genus. For example, Sheppard and Maiden (2015) demonstrated that *C. jejuni* and *C. coli* evolution was highly dependent on recombination events. These recombination events resulted in distinct lineages emerging and other large-scale genomic interspecies introgression between the two species. Gilbert et al. (2018) demonstrated that even though *C. fetus* lineages can be genetically divergent, there was WGS evidence that homologous recombination still occurred between individual *C. fetus* found in the same host. *Campylobacter* WGS has also been used in a number of studies for epidemiology characterizations including surveillance and outbreak detection, as well as phylogenetic antimicrobial resistance analyses (Biggs et al., 2011; Revez et al., 2014; Cha et al., 2016; Clark et al., 2016; Zhao et al., 2016; Llarena et al., 2017; Joensen et al., 2018).

Data from *Campylobacter* WGS have also been used to resolve and develop diagnostic assays. Jansen van Rensburg et al. (2016) used access to published WGS data to evaluate a duplex qPCR *mapA-ceuE* assay for *C. jejuni* and *C. coli* to determine how inclusive it was for all potential isolates. They used *in silico* analyses of the *mapA* and *ceuE* primer and probe sequences against 1,713 genetically diverse *C. jejuni* and *C. coli* genomes. Using the proposed primer and probe-sets, researchers demonstrated that 99.7% of the isolates tested were correctly identified. Brzozowska, et al. (2018) used a *C. jejuni* subtyping database that included data from 24,000 isolates to identify prevalent subtypes. The resulting 166 sequenced genomes were used to identify clinically associated biomarkers of *C. jejuni*. After identification of marker genes, the selected biomarkers were validated against numerous clinical and non-clinical *C. jejuni* genomes. From there, 25 genes were putatively identified as robust diagnostic biomarkers for clinical *C. jejuni* subtypes. The authors concluded that combinations of these marker genes could be used for clinical diagnostic identification of *C. jejuni* isolates that represent a significant public health risk. Furthermore, Neal-McKinney et al. (2018) used WGS in combination with mutiplex qPCR to develop a diagnostic tool for *C. jejuni* isolates that encode *cst*-*II* or *cst*-*III*-sialyltransferase. These genes are of interest because they add sialic acid to the O-antigen of lipooligosaccharide (LOS), which mimics the host’s gangliosides and has been associated with Guillain-Barre syndrome. Combining WGS to detect the genes and qPCR to screen a library of *C. jejuni* poultry field and clinical isolates along with *in silico* analyses to screen *C. jejuni* genomes revealed that the most *C. jejuni* species could produce LOS. Therefore, an assay based on these genes could be used to predict the potential of GBS from *C. jejuni* isolates. This relationship can be further exploited as the frequency of GBS-related genes may vary between isolates. The LOS gene, *wlaN,* appears to occur at a fairly low frequency (2 out of 16 *C. jejuni* and *C. coli* isolates) based on WGS profiles conducted by Cantero et al. (2018). Therefore, to further understand the relationships between copy numbers of biomarkers and the onset of GBS, more WGS data sets will need to elucidate this phenomenon. While much of the aforementioned studies are not targeted just to poultry, the patterns discovered and the tools developed will improve the food safety monitoring of poultry.

Further studies have applied WGS to poultry isolates of *Campylobacter* spp. Pendleton et al. (2013) compared PFGE (pulse-field gel electrophoresis) and *flaA* typing with WGS on a Roche 454 sequencing platform to assess discrimination capabilities among the three methods to detect *Campylobacter* from conventional and pasture flock poultry. There was no correlation between the different typing methods, but WGS appeared to be the most discriminatory and provided additional data such as the genome size and GC content. More importantly they also detected substantial evidence of genomic rearrangements within these *Campylobacter* isolates. Based on the WGS data, researchers noted that there were multiple copies of interspaced elements suggesting the potential for a high frequency of recombination events. This type of data is something likely to be missed by other non-genomic typing methods. Other WGS studies based on poultry *Campylobacter* spp. isolates have provided evidence for plasmid-mediated horizontal genetic transfer. Ghatak et al. (2017) generated a complete genomic sequence of a retail poultry *C. coli* isolate, revealing the presence of a mega-plasmid that carried several virulence factors and antibiotic resistance elements, including the plasmid-containing type IV secretion system gene.

Finally, as compared to standard detection and quantitation techniques in food safety, NGS enables researchers to evaluate other important factors for *Campylobacter* zoonosis. Evidence for horizontal spread of antibiotic resistance in *Campylobacter* spp. has been found in other *Campylobacter* isolates beyond what was discovered by Ghatak and colleagues. Florez-Cuadrado et al. (2017) conducted WGS on erythromycin resistant *C. coli* isolates from turkeys and found that *erm*(B) clustered with genes associated with aminoglycosides and tetracycline resistance. The potential for multiple horizontal transmission events was based on comparative genomic analysis where identical *erm*(B) genes were detected among *Campylobacter* from turkeys, *Streptococcus suis* from pigs, and *Enterococcus faecium* and *Clostridium difficile* from humans. Given the documented risk of antimicrobial resistance genetic element dissemination, WGS should be helpful for the large-scale surveillance and epidemiological tracking of isolates from the poultry industry. As part of the National Antimicrobial Resistance Monitoring System (NARMS), Whitehouse et al. (2018) sequenced 589 *Campylobacter* isolates from retail poultry meats. From this data set, researchers were able to identify 10 antimicrobial resistant genes. They further demonstrated a general consensus between isolate genotypes and resistance phenotypes. As more isolates are characterized by WGS, general patterns of antimicrobial resistance and novel virulence genes of interest should continue to emerge, representing the possibility of horizontal transmission. Data therein can be used to epidemiologically track the function of geographical dissemination, update poultry production and retail practices, and uncover novel relationships to improve food safety.

## Conclusions

While conventional culture methodology remains a mainstay for *Campylobacter* detection and quantitation, the consistency and reliability across various poultry production matrices are problematic. Because of the variability in *Campylobacter* species phenotypes and genotypes, more informative characterizations are needed to provide a more complete assessment of potential risk. Issues such as the existence of VBNC *Campylobacter* in different poultry production environments and the prevalence of certain virulence genes need to be taken into account when assessing and enumerating *Campylobacter* populations.

The use of high-throughput rapid microbiological methods in the poultry industry is not without challenges. New methods require sufficient validation and verification for sample type applications collected pre- or post-harvest. Generally, new methods undergo validation and certification *via* accreditation organizations such as the International Association of Analytical Communities (AOAC), wherein they are validated against “gold standard” reference methods to demonstrate equivalency or enhanced performance and fit-for-purpose use. In order to be considered attractive to the industry, new technology must be properly validated for application and use, as well as present improvements in accuracy, speed, economy, and user-friendliness. Finally, the quantitation of pathogens is becoming more important to establish baselines for assessing risk and predicting the effectiveness of overall prevention and management strategies.

Irrespective of the method chosen by the poultry industry, the specific methods and protocols need to be chosen based on food matrices, convenience, time, and cost. Immunoassays are beneficial when working with intact bacterial cells and the use of advanced proteomics should help with antigen target refinement. The improvements in PCR such as multiplex qPCR and dPCR technology should enhance their utility for routine high throughput detection assays in poultry production. The continued mining of WGS *Campylobacter* databases will allow the industry to become more comprehensive and innovative in the development of novel assays targeted to improving the sensitivity and specificity of PCR strategies. Technological improvements in WGS such as single-molecule sequencing without amplification are promising as they are capable of generating much longer reads with much less sample (Park et al., 2014; Lüth et al., 2018). However, as Lüth et al. (2018) pointed out, the standardization of wet laboratory methodology for pathogen typing, bioinformatic analyses and data storage, and mechanisms for worldwide sharing are needed, otherwise variability will make the data useless. This would seem to be particularly true for a foodborne pathogen such as *Campylobacter,* which exhibits considerable genetic variability. This variability makes it somewhat unpredictable from a transmission and detection standpoint. Enhanced and expanded WGS databases should lead to more effective surveillance and epidemiology of *Campylobacter* outbreaks and prevention.

## Author Contributions

SR and KF wrote the manuscript. WC, HP, and YY wrote parts of the manuscript and provided feedback. KF and SR provided the final edits. ZS created the figures and provided edits.

### Conflict of Interest Statement

The WC and HP are employed by Diamond V (Cedar Rapids, IA, USA).

The remaining authors declare that the research was conducted in the absence of any commercial or financial relationships that could be construed as a potential conflict of interest.

The reviewer SN and handling Editor declared their shared affiliation.

## References

[ref1] Alahi M. E. E. Mukhopadhyay S. C. (2017). Detection methodologies for pathogen and toxins: a review. Sensors 17, 1–20. 10.3390/s17081885 PMC5580025 28813028

[ref2] Alamer S. Eissa S. Chinnappan R. Zourob M. (2018). A rapid colorimetric immunoassay for the detection of pathogenic bacteria on poultry processing plants using cotton swabs and nanobeads. Microchim. Acta 185, 1–10. 10.1007/s00604-018-2696-7 29594804

[ref3] Allard M. W. (2016). The future of whole-genome sequencing for public health and the clinic. J. Clin. Microbiol. 54, 1947–1948. 10.1128/JCM.00046-16 and 10.1128/JCM.00046-16 PMC4963481 27307454

[ref4] Asal M. Özen O. Şahinler M. Potatoğlu I. (2018). Recent developments in enzyme, DNA and immuno-based biosensors. Sensors 18, 1–16. 10.3390/s18061924 PMC6021829 29899282

[ref5] Asakura M. Samosornsuk W. Taguchi M. Kobayashi K. Misawa N. Kusumoto M.. (2007). Comparative analysis of cytolethal distending toxin (cdt) genes among *Campylobacter jejuni*, *C. coli* and *C. fetus* strains. Microb. Pathog. 42, 174–183. 10.1016/j.micpath.2007.01.005, PMID: 17353111

[ref6] Asakura H. Takahashi N. Yamamoto S. Maruyama H. (2017). Draft genome sequence of *Campylobacter jejuni* CAM970 and *C. coli* CAM962, associated with a large outbreak of foodborne illness in Fukuoka, Japan, in 2016. Genome Announc. 5, e00508–e00517. 10.1128/genomeA.00508-17 28619796 PMC5473265

[ref7] Baker C. A. Rubinelli P. M. Park S. H. Ricke S. C. (2016). Immuno-based detection of shiga toxin-producing pathogenic *Escherichia coli* – a review on current approaches and potential strategies for optimization. Crit. Rev. Microbiol. 42, 656–675. 10.3109/1040841X.2015.1009824 26016737

[ref8] Baker M. (2012). Digital PCR hits its stride. Nature Meth. 9, 541–544. 10.1038/nmeth.2027

[ref9] Banowary B. Dang V. T. Sarker S. Connolly J. H. Chenu J. Groves P. (2018). Evaluation of two multiple PCR-high resolution melt curve analysis methods for differentiation of *Campylobacter jejuni* and *Campylobacter coli* intrasepecies. Avian Dis. 62, 86–93. 10.1637/11739-080417-Reg.1, PMID: 29620472

[ref10] Banowary B. Dang V. T. Sarker S. Connolly J. H. Chenu J. Groves P. (2015). Differentiation of *Campylobacter jejuni* using multiplex-PCR and high resolution melt curve analysis. PLoS One 10:e0138808. 10.1371/journal.pone.0138808 26394042 PMC4578860

[ref11] Banting G. S. Braithwaite S. Scott C. Kim J. Jeaon B. Ashbolt N.. (2016). Evaluation of various *Campylobacter*-specific quantitative PCR (qPCR) assays for detection and enumeration of *Campylobacteraceae* in irrigation water and wastewater via a miniaturized most-probable-number-qPCR assay. Appl. Environ. Microbiol. 82, 4743–4756. 10.1128/AEM.00077-16, PMID: 27235434 PMC4984289

[ref12] Barletta F. Mercado E. H. Lluque A. Ruiz J. Cleary T. G. Ochoa T. J. (2013). Multiplex real-time PCR for detection of *Campylobacter*, *Salmonella*, and *Shigella*. J. Clin. Microbiol. 51, 2822–2829. 10.1128/jcm.01397-13, PMID: 23761159 PMC3754658

[ref13] Berghman L. R. Abi-Ghanem D. Waghela S. D. Ricke S. C. (2005). Antibodies: an alternative for antibiotics? Poult. Sci. 84, 660–666. 10.1093/ps/84.4.660, PMID: 15844826 PMC7107177

[ref14] Biggs P. J. Fearnhead P. Hotter G. Mohan V. Collins-Emerson J. Kwan E. (2011). Whole-genome comparison of two *Campylobacter jejuni* isolates of the same sequence type reveals multiple loi of different ancestral lineage. PLoS One 6:e27121, 1–14. 10.1371/journal.pone.0027121 PMC3214069 22096527

[ref15] Bolton D. J. (2015). *Campylobacter* virulence and survival factors. Food Microbiol. 48, 99–108. 10.1016/j.fm.2014.11.017, PMID: 25790997

[ref16] Bonjoch X. Calvo L. Soler M. Ruiz-Rueda O. Garcia-Gil J. (2009). A new multiplexed real-time PCR assay to detect *Campylobacter jejuni*, *C. coli*, *C. lari*, and *C. upsaliensis*. Food Anal. Methods 3, 40–46. 10.1007/s12161-009-9110-3

[ref17] Bordeaux J. Welsh A. W. Agarwal S. Killiam E. Baquero M. T. Hanna J. A.. (2010). Antibody validation. BioTechniques 48, 197–209. 10.2144/000113382, PMID: 20359301 PMC3891910

[ref18] Brooks B. Mihowich W. J. G. Blais B. W. Yamazaki H. (1998). Specificity of monoclonal antibodies to *Campylobacter jejuni* lipopolysaccharide antigens. Immunol. Investig. 27, 257–265. 9730086 10.3109/08820139809070899

[ref19] Buchanan C. J. Webb A. L. Mutschall S. K. Kruczkiewicz P. Barker D. O. R. Hetman B. M. (2017). A genome-wide association study to identify diagnostic markers for human pathogenic *Campylobacter jejuni* strains. Front. Microbiol. 8, 1–9. 10.3389/fmicb.2017.01224 28713351 PMC5492696

[ref1031] Brzozowska N. Goulay J. O’Sullivan A. Buchanan F. Hannah R. Stewart A. (2018). Characterizing genetic circuit components in *E. coli* towards a *Campylobacter jejuni* biosensor. bioRxiv. 10.1101/290155

[ref20] Cantero G. Correa-Fiz F. Ronco T. Strube M. Cerdà-Cuéller M. Pedersen K. (2018). Characterization of *Campylobacter jejuni* and *Campylobacter coli* broiler isolates by whole-genome sequencing. Foodborne Pathog. Dis. 15, 145–152. 10.1089/fpd.2017.2325, PMID: 29256637

[ref21] Cao Y. Fanning S. Proos S. Jordan K. Srikumar S. (2017). A review on the applications of next generation sequencing technologies as applied to food-related microbiome studies. Front. Microbiol. 8, 1–16. 10.3389/fmicb.2017.01829 29033905 PMC5627019

[ref22] Carrino J. J. Lee H. H. (1995). Nucleic acid amplification methods. J. Microbiol. Methods 23, 3–20. 10.1016/0167-7012(95)00024-F

[ref23] Carvalho A. F. Silva D. M.., Azevedo S. S. Piatti R. M. Genovez M. E. Scarcelli E. (2013). Detection of CDT toxin genes in *Campylobacter* spp. strains isolated from broiler carcasses and vegetables in São Paulo, Brazil. Brazilian J. Microbiol. 44, 693–699. 10.1590/S1517-83822013000300005, PMID: 24516435 PMC3910176

[ref24] Castro A. G. S. A. Dorneles E. M. S. Santos E. L. S. Alves T. M. Silva G. R. Figueiredo T. C. (2018). Viability of *Campylobacter* spp. in frozen and chilled broiler carcasses according to real-time PCR with propidium monoazide pretreatment. Poult. Sci. 97, 1706–1711. 10.3382/ps/pey020 29471351

[ref25] Cha W. Mosci R. Wengert S. L. Singh P. Newton D. W. Salimnnia H. (2016). Antimicrobial susceptibility profiles of human *Campylobacter jejuni* isolates and association with phylogenetic lineages. Front. Microbiol. 7, 1–12. 10.3389/fmicb.2016.00589 27199922 PMC4845714

[ref26] Chapela M. J. Maestu A. G. Cabado A. G. (2015). Detection of foodborne pathogens by qPCR: a practical approach for food industry applications. Cogent Food Agric. 1:1013771, 1–19. 10.1080/23311932.2015.1013771

[ref27] Chen J. Park B. (2016). Recent advancements in nanobioassays and nanobiosensors for foodborne pathogenic bacteria detection. J. Food Prot. 79, 1055–1069. 10.4315/0362-028X.JFP-15-516, PMID: 27296612

[ref28] Clark C. G. Berry C. Walker M. Petkau A. Barker D. O. R. Guan C. (2016). Genomic insights from whole genome sequencing of four clonal outbreak *Campylobacter jejuni* assessed within the global *C. jejuni* population. BMC Genomics 17, 1–16. 10.1186/s12864-016-3340-8 27912729 PMC5135748

[ref29] Couturier B. A. Couturier M. R. Kalp K. J. MFisher M. A. (2013). Detection of non-*jejuni* and -*coli Campylobacter* species from stool specimens with an immunochromatographic antigen detection assay. J. Clin. Microbiol. 51, 1935–1937. 10.1128/JCM.03208-12, PMID: 23554192 PMC3716065

[ref30] Dingle K. E. Colles F. M. Ure R. Wagenaar J. A. Duim B. Bolton F. J.. (2002). Molecular characterization of *Campylobacter jejuni* clones: a basis for epidemiologic investigation. Emerg. Infect. Dis. 8, 949–955. 10.3201/eid0809.02-0122, PMID: 12194772 PMC2732546

[ref31] Eberle K. N. Kiess A. S. (2012). Phenotypic and genotypic methods for typing *Campylobacter jejuni* and *Campylobacter coli* in poultry. Poult. Sci. 91, 255–264. 10.3382/ps.2011-01414 22184452

[ref32] Eisenstein B. I. (1990). The polymerase chain reaction: a new method of using molecular genetics for medical diagnosis. N. Engl. J. Med. 322, 178–183. 10.1056/NEJM199001183220307, PMID: 2403656

[ref33] Flekna G. Stefanic P. Wagner M. Smulders F. J. M. Mozina S. S. Hein I. **(**2007). Insufficient differentiation of live and dead *Campylobacter jejuni* and *Listeria monocytogenes* cells by ethidium monoazide (EMA) compromises MA/real-time PCR. Res. Microbiol. 158, 405–412. 10.1016/j.resmic.2007.02.008, PMID: 17449228

[ref34] Floch P. Goret J. Bessède E. Lehours P. Francis Mégraud F. (2012). Evaluation of the positive predictive value of a rapid immunochromatographic test to detect *Campylobacter* in stools. Gut Path. 4:17, 1–3. 10.1186/1757-4749-4-17 PMC3538574 23206554

[ref35] Florez-Cuadrado D. Ugarte-Ruiz M. Meric G. Quesada A. Porrero M. C. Pascoe B. (2017). Genome comparison of erythromycin resistant *Campylobacter* from turkeys identifies hosts and pathways for horizontal spread of *erm*(B) genes. Front. Microbiol. 8, 1–8. 10.3389/fmicb.2017.02240 29187841 PMC5695097

[ref36] Gharst G. Oyarzabal O. A. Hussain S. K. (2013). Review of current methodologies to isolate and identify *Campylobacter* spp. from foods. J. Microbiol. Methods 95, 84–92. 10.1016/j.mimet.2013.07.014, PMID: 23899774

[ref37] Ghatak S. He Y. Reed S. Strobaugh T. Jr. Irwin P. (2017). Whole genome sequencing and analysis of *Campylobacter coli* YH502 from retail chicken reveals a plasmid-borne type VI secretion system. Genomic Data 11, 128–131. 10.1016/j.gdata.2017.02.005, PMID: 28217442 PMC5302137

[ref38] Giesendorf B. A. Quint W. G. Henkens M. H. Stegeman H. Huf F. A. Hiesters H. G. (1992). Rapid and sensitive detection of *Campylobacter* spp. in chicken products using the polymerase chain reaction. Appl. Environ. Microbiol. 58, 3804–3808. PMID: 1282312 10.1128/aem.58.12.3804-3808.1992 PMC183185

[ref39] Gilbert M. J. Duim B. van der Graaf-van Bloois L. Wagenaar J. A. Zomer A. L. (2018). Homolgous recombination between genetically divergent *Campylobacter fetus* lineages supports host-associated speciation. Genome Biol. Evol. 10, 716–722. 10.1093/gbe/evy048, PMID: 29608720 PMC5830970

[ref40] Granato P. A. Chen L. Holiday I. Rawling R. A. Novak-Weekley S. M. Quinlan T.. (2010). Comparison of Premier CAMPY Enzyme Immunoassay (EIA), ProSpecT *Campylobacter* EIA, and ImmunoCard STAT! CAMPY Tests with culture for laboratory diagnosis of *Campylobacter* enteric infections. J. Clin. Microbiol. 48, 4022–4027. 10.1128/JCM.00486-10, PMID: 20810765 PMC3020833

[ref41] Guerry P. Logan S. M. Thornton S. (1990). Genomic organization and expression of *Campylobacter* flagellin genes. J. Bacteriol. 172, 1853–1860. 10.1128/jb.172.4.1853-1860.1990, PMID: 2318805 PMC208679

[ref42] Hannson I. Sandberg M. Habib I. Lowman R. Evgvall E. O. (2016). Knowledge gaps in control of *Campylobacter* for prevention of campylobacteriosis. Tansbound Emerg. Dis. 65, 30–48. 10.1111/tbed.12870 29663680

[ref43] He Y. Yao X. Gunther N. W. Xie Y. Tu S. I. Shi X. (2010). Simultaneous detection and differentiation of *Campylobacter jejuni*, *C. coli*, and *C. lari* in chickens using a multiplex real-time PCR assay. Food Anal. Methods 3, 321–329. 10.1007/s12161-010-9136-6

[ref44] Heo S. A. Nannapaneni R. Johnson M. G. Park J. S. Seo K. H. (2009). Production and characterization of a monoclonal antibody to *Campylobacter jejuni*. J. Food Prot. 72, 870–875. 10.4315/0362-028X-72.4.870, PMID: 19435241

[ref1030] Higuchi R. Fockler C. Dollinger G. Watson R. (1993). Kinetic PCR Analysis: Real-time monitoring of DNA amplification reactions. Nat. Biotechnol. 11, 1026–1030. PMID: 7764001 10.1038/nbt0993-1026

[ref45] Hill W. E. (1996). The polymerase chain reaction: applications for the detection of foodborne pathogens. Crit. Rev. Food Sci. Nutr. 36, 123–173. 8747102 10.1080/10408399609527721

[ref46] Hofreiter D. Novik V. Galán J. E. (2008). Metabolic diversity in *Campylobacter jejuni* enhances specific tissue colonization. Cell Host Microbe 4, 425–433. 10.1016/j.chom.2008.10.002, PMID: 18996343

[ref47] Horrocks S. M. Anderson R. C. Nisbet D. J. Ricke S. C. (2009). Incidence and ecology of *Campylobacter jejuni* and *coli* in animals. Anaerobe 15, 8–25. 10.1016/j.anaerobe.2008.09.001 18849005

[ref48] Huang H. Brooks B. W. Lowman R. Carillo C. D. (2015). *Campylobacter* species in animal, food, and environmental sources, and relevant testing programs in Canada. Can. J. Microbiol. 61, 701–721. 10.1139/cjm-2014-0770, PMID: 26422448

[ref49] Huang J. Yang G. Meng W. Wu L. Zhu A. Jiao X. (2010). An electrochemical impedimetric immunosensor for label-free detection of *Campylobacter jejuni* in diarrhea patients’ stool based on O-carboxymethylchitosan surface modified Fe_3_O_4_ nanoparticles. Biosens. Bioelectron. 25, 1204–1211. 10.1016/j.bios.2009.10.036, PMID: 19932018

[ref50] Huggett J. F. Cowen S. Foy C. A. (2015). Considerations for digital PCR as an accurate molecular diagnostic tool. Clin. Chem. 61, 79–88. 10.1373/clinchem.2014.221366 25338683

[ref51] Humphrey T. O’Brien S. Madsen M. (2007). *Campylobacters* as zoonotic pathogens: a food production perspective. Int. J. Food Microbiol. 117, 237–257. 10.1016/j.ijfoodmicro.2007.01.006, PMID: 17368847

[ref52] Huq M. Gonis G. Istivan T. (2014). Development and evaluation of a multiplex PCR for the detection of *Campylobacter concisus* and other *Campylobacter* spp. from gastroenteritis cases. Open J. Med. Microbiol. 4, 29–37. 10.4236/ojmm.2014.41005

[ref53] Inglis G. D. Kalischuk L. D. (2003). Use of PCR for direct detection of *Campylobacter* species in bovine species. Appl. Environ. Microbiol. 69, 3435–3447. 10.1128/AEM.69.6.3435-3447.2003, PMID: 12788747 PMC161499

[ref1034] Jackson C. J. Fox A. J. Jones D. M. (1996a). A novel polymerase chain reaction assay for the detection of speciation of thermophilic *Campylobacter* spp. J. Appl. Microbiol. 81, 467–473. 10.1111/j.1365-2672.1996.tb03534.x 8939024

[ref1035] Jackson C. J. Fox A. J. Warening D. R. A. Hutchinson D. N. Jones D. M. (1996b). The application of genotyping techniques to the epidemiological analysis of *Campylobacter jejuni*. Epidemiol. Infect. 117, 233–244. 8870620 10.1017/s0950268800001400 PMC2271704

[ref54] Janda A. Bowen A. Greenspan N. S. Casadevall A. (2016). Ig constant region effects on variable region structure and function. Front. Microbiol. 7, 1–10. 10.3389/fmicb.2016.00022 26870003 PMC4740385

[ref55] Jansen van Rensburg M. Swift C. Cody A. J. Jenkins C. Maiden M. C. J. (2016). Exploiting bacterial whole-genome sequencing data for evaluation of diagnostic assays: *Campylobacter* species identification as a case study. J. Clin. Microbiol. 54, 2882–2890. 10.1128/JCM.01797-16, PMID: 27733632 PMC5121375

[ref56] Joensen K. G. Kuhn K. G. Muller L. Bjorkman J. T. Torpdahl M. Engberg J. (2018). Whole genome sequencing of *Campylobacter jejuni* isolated from Danish routine stool samples reveals surprising degree of clustering. Clin. Microbiol. Infect. 24, 201.e5–201.e8. 10.1016/j.cmi.2017.07.026 28782648

[ref57] Josefsen M. H. Jacobsen N. R. Hoorfar J. (2004). Enrichment followed by quantitative PCR both for rapid detection and as a tool for quantitative risk assessment of food-borne thermotolerant campylobacters. Appl. Environ. Microbiol. 70, 3588–3592. 10.1128/AEM.70.6.3588-3592.2004, PMID: 15184161 PMC427791

[ref58] Josefsen M. H. Löfström C. Hansen T. B. Chtistensen L. S. Olsen J. E. Hoorfar J. (2010). Rapid quantitation of viable *Campylobacter* bacteria on chicken carcasses, using real-time PCR and propidium monazide treatment, as a tool for quantitative risk assessment. Appl. Environ. Microbiol. 76, 5097–5104. 10.1128/AEM.00411-10, PMID: 20562292 PMC2916463

[ref59] Kamei K. Kawabata H. Asakura M. Samosornsuk W. Hinenoya A. Nakagawa S.. (2016). A cytolethal distending toxin gene-based multiplex PCR assay for *Campylobacter jejuni*, *C. fetus*, *C. coli*, *C. upsaliensis*, *C. hyointestinalis*, and *C. lari*. Jpn. J. Infect. Dis. 69, 256–258. 10.7883/yoken.JJID.2015.182, PMID: 26255737

[ref60] Kawase J. Kurosaki M. Kawakami Y. Kashimoto T. Tsunomori Y. Sato K.. (2014). Comparison of two methods of bacterial DNA extraction from fecal samples contaminated with *Clostridium perfringens*, *Staphylococcus aureus*, *Salmonella Typhimurium*, *Campylobacter jejuni*. Jpn. J. Infect. 67, 441–446. 10.7883/yoken.67.441, PMID: 25410559

[ref61] Kawatsu K. Kumeda Y. Taguchi M. Yamazaki-Matsune W. Kanki M. Inoue K. (2008). Development and evaluation of immunochromatographic assay for simple and rapid detection of *Campylobacter jejuni* and *Campylobacter coli* in human stool specimens. J. Clin. Microbiol. 46, 1226–1231. 10.1128/JCM.02170-07, PMID: 18256225 PMC2292965

[ref62] Kim S. A. Park S. H. Lee S. I. Owens-Hanning C. Ricke S. C. (2017a). Assessment of chicken carcass microbiome responses during processing in the presence of commercial antimicrobials using a next generation sequencing approach. Sci. Rep. 7:43354. 10.1038/srep43354 28230180 PMC5322484

[ref63] Kim S. A. Park S. H. Lee S. I. Ricke S. C. (2017b). Development of a rapid method to quantify *Salmonella* Typhimurium using a combination of MPN with qPCR and a shortened time incubation. Food Microbiol. 65, 7–18. 10.1016/j.fm.2017.01.013 28400022

[ref64] Klena J. D. Parker C. T. Knibb K. Ibbitt J. C. Devane P. M. Horn S. T.. (2004). Differentiation of *Campylobacter coli*, *Campylobacter jejuni*, *Campylobacter lari*, and *Campylobacter upsaliensis* by a multiplex PCR developed from the nucleotide sequence of the lipd A gene lpxA. J. Clin. Microbiol. 42, 5549–5557. 10.1128/JCM.42.12.5549-5557.2004, PMID: 15583280 PMC535264

[ref65] Kralik P. Ricchi M. (2017). A basic guide to real time PCR in microbial diagnostics: definitions, parameters, and everything. Front. Microbiol. 8, 1–9. 10.3389/fmicb.2017.00108 28210243 PMC5288344

[ref66] Krüger N. J. Buhler C. Iwoba A. N. Huber I. Ellerbroek L. Appel B.. (2014). “Limits of control” – crucial parameters for a reliable quantification of viable *Campylobacter* by real-time PCR. PLoS One 9:e88108. 10.1371/journal.pone.0088108, PMID: 24505398 PMC3914927

[ref67] Lahti E. Löfdahl M. Ågren J. Hansson I. Olsson Engvall E. (2017). Confirmation of a campylobacteriosis outbreak associated with chicken liver pâté using PFGE and WGS. Zoonoses Public Health 64, 14–20. 10.1111/zph.12272, PMID: 27334628

[ref68] Lamoureux M. Mackay A. Messier S. Fliss I. Blais B. W. Holley R. A.. (1997). Detection of *Campylobacter jejuni* in food and poultry viscera using immunomagentic separation and microtitre hybridization. J. Appl. Microbiol. 83, 641–651. 10.1046/j.1365-2672.1997.00273.x, PMID: 9418026

[ref69] Lawton S. J. Weis A. M. Byrne B. A. Fritz H. Taff C. C. Townsend A. K.. (2018). Comparative analysis of *Campylobacter* isolates from wild birds and chickens using MALDI-TOF MS, biochemical testing, and DNA sequencing. J. Vet. Diagn. Investig. 30, 354–361. 10.1177/1040638718762562, PMID: 29528812 PMC6505823

[ref70] Leekitcharoenphon P. Garcia-Graells C. Botteldoorn N. Dierick K. Kempf I. Olkkola S. (2018). Comparative genomics of quinolone-resistant and susceptible *Campylobacter jejuni* of poultry origin from major poultry producing European countries (GENCAMP). EFSA Supporting Publications 15:1398E.

[ref71] Leskinen S. D. Lim D. V. (2008). Rapid ultrafiltration concentration and biosensor detection of enterococci from large volumes of Florida recreational water. Appl. Environ. Microbiol. 7415, 4792–4798. 10.1128/AEM.00052-08 PMC2519343 18515479

[ref72] Linton D. Owen R. J. Stanley J. (1996). Rapid identification by PCR of the genus *Campylobacter* and of five *Campylobacter* species enteropathogenic for man and animals. Res. Microbiol. 147, 707–718. 10.1016/S0923-2508(97)85118-2, PMID: 9296105

[ref73] Linton D. Lawson A. J. Owen R. J. Stanley J. P. C. R. (1997). PCR detection, identification to species level, and fingerprinting of *Campylobacter jejuni* and *Campylobacter coli* direct from diarrheic samples. J. Clin. Microbiol. 35, 2568–2572. PMID: 9316909 10.1128/jcm.35.10.2568-2572.1997 PMC230012

[ref74] Llarena A. K. Taboada E. Rossi M. (2017). Whole-genome sequencing in epidemiology of *Campylobacter jejuni* infections. J. Clin. Microbiol. 55, 1269–1275. 10.1128/JCM.00017-17, PMID: 28249998 PMC5405246

[ref75] Logan J. M. J. Edwards K. J. Saunders N. A. Stanley J. (2001). Rapid identification of *Campylobacter* spp. by melting peak analysis of biprobes in real-time PCR. J. Clin. Microbiol. 39, 2227–2232. 10.1128/JCM.39.6.2227-2232.2001, PMID: 11376061 PMC88115

[ref76] Loge F. J. Thompson D. E. Call D. R. (2002). PCR detection of specific pathogens in water: a risk-based analysis. Environ. Sci. Technol. 3612, 2754–2759. 10.1021/es015777m 12099475

[ref77] Lund M. Nordentoft S. Pedersen K. Madsen M. (2004). Detection of *Campylobacter* spp. in chicken fecal samples by real-time PCR. J. Clin. Microbiol. 42, 5125–5132. 10.1128/JCM.42.11.5125-5132.2004, PMID: 15528705 PMC525216

[ref78] Lüth S. Kleta S. Dahouk S. A. (2018). Whole genome sequencing as a typing tool for foodborne pathogens like *Listeria monocytogenes*—the way towards global harmonization and data exchange. Trends Food Technol. Technol. 73, 67–75. 10.1016/j.tifs.2018.01.008

[ref79] Maciorowski K. G. Herrera P. Jones F. T. Pillai S. D. Ricke S. C. (2006). Cultural and immunological detection methods for detection of *Salmonella* spp. in animal feeds: a review. Veterinary Res. Comm. 30, 127–137. 10.1007/s11259-006-3221-8, PMID: 16400599

[ref80] Mackay I. M. (2004). Real-time PCR in the microbiology laboratory. Clin. Microbiol. Infect. 10, 190–212. 10.1111/j.1198-743X.2004.00722.x, PMID: 15008940

[ref81] Mäesaar M. Meremäe K. Ivanova M. Roasto M. (2018). Antimicrobial resistance and multilocus sequence types of *Campylobacter jejuni* isolated from Baltic broiler chicken meat and Estonian human patients. Poult. Sci. 97, 3645–3651. 10.3382/ps/pey219, PMID: 29850847

[ref82] Mandal P. K. Biswas A. K. Choi K. Pal U. K. (2011). Methods for rapid detection of foodborne pathogens: an overview. Am. J. Food Technol. 6, 87–102. 10.3923/ajft.2011.87.102

[ref83] Manfreda G. De Cesare A. (2005). *Campylobacter* and *Salmonella* in poultry and poultry products: hows and whys of molecular typing. World’s Poultry Sci. J. 61, 185–197. 10.1079/WPS200448

[ref84] Masdor N. A. Altintas Z. Tothill I. E. (2017). Surface plasmon resonance immunosensor for the detection of *Campylobacter jejuni*. Chem. Aust. 5, 1–15. 10.3390/chemosensors5020016

[ref85] Masdor N. A. Altintas Z. Tothill I. E. (2016). Sensitive detection of *Campylobacter jejuni* using nanoparticles enhanced QCM sensor. Biosens. Bioelectron. 78, 328–336. 10.1016/j.bios.2015.11.033, PMID: 26649490

[ref86] Mehla K. Ramana J. (2017). Surface proteome mining for identification of potential vaccine candidates against *Campylobacter jejuni*: an in silico approach. Funct. Integr. Genomics 17, 27–37. 10.1007/s10142-016-0530-z 27778110

[ref87] Metzker M. L. (2010). Sequencing technologies–the next generation. Nature Revs. 11, 31–46. 10.1038/nrg2626, PMID: 19997069

[ref88] Miller W. G. Parker C. T. Heath S. Lastovica A. J. (2007). Identification of genomic differences between *Campylobacter jejuni* subsp. *jejuni* and *C. jejuni* subsp. *doylei* at the *nap* locus leads to the development of a *C. jejuni* subspeciation multiplex PCR method. BMC Microbiol. 7, 1–8. 10.1186/1471-2180-7-11 17328805 PMC1820782

[ref89] Myers A. L. Jackson M. A. Selvarangan R. (2011). False-positive results of *Campylobacter* rapid antigen testing. Pediatr. Infect. Dis. J. 30:542. 10.1097/INF.0b013e31821524db, PMID: 21587030

[ref90] Nachamkin I. Hart A. M. (1986). Common and specific epitopes of *Campylobacter* flagellin recognized by monoclonal antibodies. Appl. Environ. Microbiol. 53, 438–440. 10.1128/iai.53.2.438-440.1986 PMC260896 2426201

[ref91] Neal-McKinney J. M. Liu K. C. Jinneman K. C. Wu W.-H. Rice D. H. (2018). Whole genome sequencing and multiplex qPCR methods to identify *Campylobacter jejuni* encoding *cst-II* or *cst-III* sialyltransferase. Front. Microbiol. 9, 1–8. 10.3389/fmicb.2018.00408 29615986 PMC5865068

[ref92] Nogva H. K. Bergh A. Holck A. Rudi K. (2000). Application of the 5′-nuclease PCR assay in evaluation and development of methods for quantitative detection of *Campylobacter jejuni*. Appl. Environ. Microbiol. 66, 4029–4036. 10.1128/AEM.66.9.4029-4036.2000, PMID: 10966425 PMC92255

[ref93] Nzuma R. M. Liu F. Grant I. R. (2018). Generation and characterization of a novel recombinant scFv antibody specific for *Campylobacter jejuni*. Appl. Microbiol. Biotechnol. 102, 4873–4885. 10.1007/soo253-018-8949-x, PMID: 29627856 PMC5953994

[ref94] Oakley B. B. Morales C. A. Line J. E. Seal B. S. Hiett K. L. (2012). Application of high-throughput sequencing to measure the performance of commonly used selective cultivation methods for the foodborne pathogen *Campylobacter*. FEMS Microbiol. Ecol. 79, 327–336. 10.1111/j.1574-6941.2011.01219.x, PMID: 22092388

[ref95] O’Connell P. J. O’Sullivan C. K. Guibault G. G. (2000). Biosensors for food analysis. Irish J. Agric. Food Res. 39, 321–329.

[ref96] O’Kane P. M. Connerton I. F. (2017). Characterisation of aerotolerant forms of a robust chicken colonizing *Campylobacter coli*. Front. Microbiol. 8:513. 10.3389/fmicb.2017.00513, PMID: 28396658 PMC5366326

[ref97] On S. L. Brandt S. M. Corneluis A. J. Fusco V. Quero G. M. Maćkiw E. (2013). PCR revisited: a case for revalidation of PCR assays for microorganisms using identification of *Campylobacter* species as an example. Qual. Assurance Safety Crops Foods 5, 49–62. 10.3920/QAS2012.0158

[ref98] On S. L. W. Holmes B. Sackin M. J. (1996). A probability matrix for the identification of campylobacters, helicobacters and allied taxa. J. Appl. Bacteriol. 81, 425–432. PMID: 8896353 10.1111/j.1365-2672.1996.tb03529.x

[ref99] Oyarzabal O. A. Battie C. (2012). “Chapter 13. Immunological methods for the detection of *Campylobacter* spp.–current applications and potential use in biosensors” in Trends in immunolabelled and related techniques. ed. Abuelzein E. (Rijeka, Croatia: InTech), 203–226.

[ref100] Oyofo B. A. Thornton S. A. Burr D. H. Trust T. J. Pavlovskis O. R. Guerry P. (1992). Specific detection of *Campylobacter jejuni* and *Campylobacter coli* by using polymerase chain reaction. J. Clin. Microbiol. 30, 2613–2619. PMID: 1400961 10.1128/jcm.30.10.2613-2619.1992 PMC270487

[ref101] Pacholewicz E. Swart A. Lipman L. J. A. Wagenaar J. A. Havelaar A. H. Duim B. (2013). Propidium monazide does not fully inhibit the detection of dead *Campylobacter* on broiler chicken carcasses by qPCR. J. Microbiol. Methods 95, 32–38. 10.1016/j.mimet.2013.06.003, PMID: 23811205

[ref102] Papic B. Pate M. Henigman U. Zajc U. Gruntar I. Biazzo M. (2017) New approaches on qunatifiaction of *Campylobacter jejuni* in poultry samples: the use of digital PCR and real-time PCR against the ISO standard plate count method. Front. Microbiol. 8, 1–13. 10.3389/fmicb.2017.00331 28303130 PMC5332403

[ref103] Park S. H. Aydin M. Khatiwara A. Dolan M. C. Gilmore D. F. Bouldin J. L.. (2014). Current and emerging technologies for rapid detection and characterization of *Salmonella* in poultry and poultry products. Food Microbiol. 38, 250–262. 10.1016/j.fm.2013.10.002, PMID: 24290649

[ref104] Park S. H. Hanning I. Jarquin R. Moore P. Donoghue D. J. Donoghoue A. M. (2011) Multiplex PCR assay for the detection and quantification of *Campylobacter* spp., *Escherichia coli* O157:H7, and *Salmonella* serotypes in water samples. FEMS Microbiol. Letters 316, 7–15. 10.1111/j.1574-6968.2010.02188.x 21204924

[ref105] Parkhill J. Wren B. W. Mungall K. Ketley J. M. Churcher C. Basham D.. (2000). The genome sequence of the food-borne pathogen *Campylobacter jejuni* reveals hypervariable sequences. Nature 403, 665–668. 10.1038/35001088, PMID: 10688204

[ref106] Pendleton S. Hanning I. Biswas D. Ricke S. C. (2013). Evaluation of whole-genome sequencing as a genotyping tool for *Campylobacter jejuni* in comparison with pulsed-field gel electrophoresis and *flaA* typing. Poult. Sci. 92, 573–580. 10.3382/ps.2012-02695, PMID: 23300325

[ref107] Pennisi E. (2017). Pocket-sized sequencers start to pay off big. Science 356, 572–573. 10.1126/science.356.6338.572, PMID: 28495708

[ref108] Pennisi E. (2016). Pocket DNA sequencers make real-time diagnostics a reality–Advances in accuracy of nanopore sequencing help pave the way for on-the-spot DNA tests. Science 351, 800–801. 10.1126/science.351.6275.800, PMID: 26912872

[ref109] Persson S. Olsen K. E. P. (2005). Multiplex PCR for identification of *Campylobacter coli* and *Campylobacter jejuni* from pure cultures and directly on stool samples. J. Med. Microbiol. 54, 1043–1047. 10.1099/jmm.0.46203-0, PMID: 16192435

[ref110] Pontiroli A. Travis E. R. Sweeney F. P. Porter D. Gaze W. H. Mason S. (2011). Pathogen quantitation in complex matrices: a multi-operator comparison of DNA extraction methods with a novel assessment of PCR inhibition. PLoS One 6:e17916. 10.1371/journal.pone.0017916 21448453 PMC3063169

[ref111] Preiner J. Kodera N. Tang J. Ebner A. Brameshuber M. Blaas D. (2014). IgGs are made for walking on bacterial and viral surfaces. Nature Comm. 5:4394. 10.1038/ncomms5394 25008037

[ref112] Qian H. Pang E. Du Q. Chang J. Dong J. Toh S. L.. (2008). Production of a monoclonal antibody specific for the major outer membrane protein of *Campylobacter jejuni* and characterization of the epitope. Appl. Environ. Microbiol. 74, 833–839. 10.1128/AEM.01559-07, PMID: 18065632 PMC2227716

[ref113] Raja S. Rao A. Babu N. Thamizhannal (2016). Detection of emerging food pathogens in chicken meat using multiplex polymerase chain reaction. J. Agric. Sci. 8, 217–225. 10.5539/jas.v8n9p217

[ref114] Rasmussen H. N. Olsen J. E. Jørgensen K. Rasmussen O. F. (1996). Detection of *Campylobacter jejuni* and *Camp. coli* in chicken faecal samples by PCR. Lett. Appl. Microbiol. 23, 363–366. 10.1111/j.1472-765X.1996.tb00209.x, PMID: 8987720

[ref115] Regnath T. Ignatius R. (2014). Accurate detection of *Campylobacter* spp. antigens by immunochromatography and enzyme immunoassay in routine microbiological laboratory. European J. Microbiol. Immunol. 4, 156–158. 10.1556/EUJMI-D-14-00018, PMID: 25215191 PMC4160794

[ref116] Reis L. P. Meneze L. D. M. Lima G. K. de Souza Santos E. L. Dorneles E. M. S. de Assis D. C. S.. (2018). Detection of *Campylobacter* spp. in chilled and frozen broiler carcasses comparing immunoassay, PCR and real time PCR methods. Ciênc. Rural, Santa Maria 48:e20161034. 10.1371/journal.pone.0208488, PMID: 30540821 PMC6291294

[ref117] Reshetilov A. N. (2005) Microbial, enzymatic, and immune biosensors for ecological monitoring and control of biotechnological processes. Appl. Biochem. Microbiol. 41, 442–449. 10.1007/s10438-005-0079-4 16240647

[ref118] Revez J. Llarena A.-K. Schott T. Kuusi M. Hakkinen M. Kivisto R. (2014). Genome analysis of *Campylobacter jejuni* strains isolated from a waterborne outbreak. BMC Genomics 15, 1–8. www.biomedcentral.com/1471-2164/15/768. 10.1186/1471-2164-15-768 25196593 PMC4168118

[ref119] Rokney A. Valinsky L. Moran-Gilad J. Vranckx K. Agmon V. Weinberger M. (2018). Genomic epidemiology of *Campylobacter jejuni* transmission in Isreal. Front. Microbiol. 9:2432. 10.3389/fmicb.2018.02432 30386311 PMC6198274

[ref120] Rodrigues R. C. Haddad N. Chevret D. Cappelier J. M. Tresse O. (2016). Comparsion of proteomics profiles of *Campylobacter jejuni* strain Bf under microaerobic and aerobic conditions. Front. Microbiol. 7, 1–12. 10.1002/cpt.1328, PMID: 27790195 PMC5061731

[ref121] Ronholm J. (2018). Editorial: game changer–next generation sequencing and its impact on food microbiology. Front. Microbiol. 9, 1–3. 10.3389/fmicb.2018.00363 29593663 PMC5854679

[ref122] Roop II R. M. Smibert R. M. Johnson J. L. Krieg N. R. (1984). Differential characteristics of catalase–positive campylobacters correlated with DNA homology groups. Can. J. Microbiol. 30, 938–951. 10.1139/m84-147, PMID: 6478314

[ref1033] Rothrock Jr. M. Hiett K. L. Kiepper B. H. Ingram K. Hinton A. (2013). Commercial poultry processing water samples using droplet digital PCR. Adv. Microbiol. 3, 403–411.

[ref123] Rothrock Jr. M. J. Hiett K. L. Kiepper B. H. Ingram K. Hinton A. (2015). Quantification of zoonotic bacterial pathogens within commercial poultry processing water samples using droplet digital PCR. Adv. Microbiol. 3, 403–411. 10.4236/aim.2013.35055

[ref124] Rudi K. Moen B. Drømtorp S. M. Holck A. L. (2005). Use of ethidium monoazide and PCR in combination for quantification of viable and dead cells in complex samples. Appl. Environ. Microbiol. 71, 1018–1024. 10.1128/AEM.71.2.1018-1024.2005, PMID: 15691961 PMC546808

[ref125] Rudi K. Høidal H. K. Katla T. Johansen B. K. Nordal J. Jakobsen K. S. (2004). Direct real-time PCR quantification of *Campylobacter jejuni* in chicken fecal and cecal samples by integrated cell concentration and DNA purification. Appl. Environ. Microbiol. 70, 790–797. 10.1128/AEM.70.2.790-797.2004, PMID: 14766556 PMC348921

[ref126] Sacher J. C. Yee E. Szymanski C. M. Miller W. G. (2018). Complete genome sequences of three *Campylobacter jejuni* phage-propagating strains. Genome Announc. 6, e00514–e00518. 10.1128/genomeA.00514-18 29903820 PMC6003733

[ref127] Sails A. D. Bolton F. J. Fox A. J. Wareing D. R. Greenway D. L. (2002). Detection of *Campylobacter jejuni* and *Campylobacter coli* in environmental waters by PCR enzyme-linked immunosorbent assay. Appl. Environ. Microbiol. 68, 1319–1324. 10.1128/AEM.68.3.1319-1324.2002, PMID: 11872483 PMC123752

[ref128] Scallan E. Hoekstra R. M. Angulo F. J. Tauxe R. V. Widdowson M. A. Roy S. L.. (2011). Foodborne illness acquired in the United States-major pathogens. Emerg. Infect. Dis. 17, 7–15. 10.3201/eid1701.P11101, PMID: 21192848 PMC3375761

[ref129] Seinige D. Krischek C. Klein G. Kehrenberg C. (2014). Comparative analysis and limitations of ethidium monoazide and propidium treatments for the differentiation of viable and nonviable *Campylobacter* cells. Appl. Environ. Microbiol. 80, 2186–2192. 10.1128/AEM.03962-13, PMID: 24487529 PMC3993131

[ref130] Sekse C. Holst-Jensen A. Dobrindt U. Johannessen G. S. Li W. Spilsberg B. (2017). High throughput sequencing for detection of foodborne pathogens. Front. Microbiol. 8, 1–26. 10.3389/fmicb.2017.02029 29104564 PMC5655695

[ref131] Sheppard S. K. Maiden M. C. J. (2015). The evolution of *Campylobacter jejuni* and *Campylobacter coli*. Cold Spring Harb. Perspect. Biol. 7:a018119, 1–13. www.cshperspectives.org. 10.1101/cshperspect.a018119 PMC4526750 26101080

[ref132] Steele M. Gyles C. Chan V. L. Odumeru J. (2002). Monoclonal antibodies specific for hippurate hydrolase of *Campylobacter jejuni*. J. Clin. Microbiol. 40, 1080–1082. 10.1128/jcm.40.3.1080-1082.2002, PMID: 11880445 PMC120243

[ref133] Taboada E. N. Graham M. R. Carriço J. A. Van Domselaar G. (2017). Food safety in the age of next generation sequencing, bioinformatics, and open data access. Front. Microbiol. 8, 1–10. 10.3389/fmicb.2017.00909 28588568 PMC5440521

[ref134] Taboada E. N. Clark C. G. Sproston E. L. Carrillo C. D. (2013). Current methods for molecular typing of *Campylobacter* species. J. Microbiol. Methods 95, 24–31. 10.1016/j.mimet.2013.07.007 23871858

[ref135] Taveirne M. E. Dunham D. T. Perault A. Beauchamp J. M. Huynh S. Parker C. T. (2017). Complete annotated genome sequences of three *Campylobacter jejuni* strains isolated from naturally colonized farm-raised chickens. Genome Announc. 5, e01407–e01416. 10.1128/genomeA.01407-16 28126931 PMC5270690

[ref136] Thépault A. Rose V. Quesne S. Poezevara T. Béven V. Hirchaud E. (2018). Ruminant and chicken: important sources of campylobacteriosis in France despite a variation of source attribution in 2009 and 2015. Sci. Rep. 8: 9305. 10.1038/s41598-018-27558-z 29915208 PMC6006168

[ref137] Tholozan J. L. Cappelier J. M. Tissier J. P. Delattre G. Federighi M. (1999). Physiological characterization of viable-but-nonculturable *Campylobacter jejuni* cells. Appl. Environ. Microbiol. 65, 1110–1116. 10049870 10.1128/aem.65.3.1110-1116.1999 PMC91151

[ref138] Toplak N. Kovač M. Piskernik S. Možina S. S. Jeršek B. (2012). Detection and quantification of *Campylobacter jejuni* and *Campylobacter coli* using real-time multiplex PCR. J. Appl. Microbiol. 112, 752–764. 10.1111/j.1365-2672.2012.05235.x, PMID: 22256961

[ref139] Turonova H. Haddad N. Hernould M. Chevret D. Paziarova J. Tresse O. (2017). Profiling of *Campylobacter jejuni* proteome in exponential and stationary phase of growth. Front. Microbiol. 8, 1–12. 10.3389/fmicb.2017.00913 28572800 PMC5435804

[ref140] Ugarte-Ruiz M. Wassenaar T. M. Gómez-Barrero S. Porrero M. C. Navarro-Gonzalez N. Dominguez L. (2013). The effect of different isolation protocols on detection and molecular characterization of *Campylobacter* from poultry. Lett. Appl. Microbiol. 57, 427–435. 10.1111/lam.12130, PMID: 23837671

[ref141] Uyttendaele M. Schukkink R. van Gemen B. Debevere J. (1995). Detection of *Campylobacter jejuni* added to foods by using a combined selective enrichment and nucleic acid sequence-based amplification (NASBA). Int. J. Food Microbiol. 61, 1341–1347. PMID: 7747955 10.1128/aem.61.4.1341-1347.1995 PMC167389

[ref142] Välimaa A.-L. Tilsala-Timisjärvi A. Virtanen E. (2015). Rapid detection and identification methods for *Listeria monocytogenes* in the food chain: a review. Food Control 55, 103–114. 10.1016/j.foodcont.2015.02.037

[ref143] Villamizar-Rodríguez G. Fernández J. Marín L. Muñiz J. González I. Lombó F. (2015). Multiplex detection of nine food-borne pathogens by mPCR and capillary electrophoresis after using a universal pre-enrichment medium. Front. Microbiol. 6, 1–16. 10.3389/fmicb.2015.01194, PMID: 26579100 PMC4630290

[ref144] Wang G. Clark C. G. Taylor T. M. Pucknell C. Barton C. Price L.. (2002). Colony multiplex PCR assay for identification and differenctiation of *Campylobacter jejuni*, *C. coli*, *C. upsaliensis*, and *C. fetus* subsp. *fetus*. J. Clin. Microbiol. 40, 4744–4747. 10.1128/JCM.40.12.4744-4747.2002, PMID: 12454184 PMC154608

[ref145] Wassenaar T. M. Newell D. G. (2000). Genotyping of *Campylobacter* spp. Appl. Environ. Microbiol. 66, 1–9. 10.1128/AEM.66.1.1-9.2000, PMID: 10618195 PMC91777

[ref146] Wassenaar T. M. Geilhausen B. Newell D. G. (1998). Evidence of genomic instability in *Campylobacter jejuni* isolated from poultry. Appl. Environ. Microbiol. 64, 1816–1821. PMID: 9572956 10.1128/aem.64.5.1816-1821.1998 PMC106235

[ref147] Wei D. Oyarzabal O. A. Huang T.-S. Balasubramanian S. Sista S. Simonian A. L. (2007). Development of a surface plasmon resonance biosensor for the identification of *Campylobacter jejuni*. J. Microbiol. Methods 69, 78–85. 10.1016/j.mimet.2006.12.002, PMID: 17258830

[ref148] Whitehouse C. A. Young S. Li C. Hsu C.-H. Martin G. (2018). Use of whole-genome sequencing for *Campylobacter* surveillance from NARMS retail poultry in the United States in 2015. Food Microbiol. 73, 122–128. 10.1016/j.fm.2018.01.018, PMID: 29526197

[ref149] Willner I. (2002). Biomaterials for sensors, fuel cells, and circuitry. Science 298, 2407–2408. 10.1126/science.298.5602.2407, PMID: 12493919

[ref150] Wilson D. L. Rathinam V. A. K. Qi W. Wick L. M. Landgraf J. Bell J. A., et al. (2010). Genetic diversity in *Campylobacter jejuni* is associated with differential colonization of broiler chickens and C57BL/6J IL10-deficient mice. Microbiology 156, 2046–2057. 10.1099/mic.0.035717-0 20360176 PMC3068676

[ref151] Wittwer C. T. Herrmann M. G. Gundry C. N. Elenitoba-Johnson K. S. J. (2001). Realtime multiplex PCR assays. Methods 25, 430–442. 10.1006/meth.1265, PMID: 11846612

[ref152] Yamazaki-Matsune W. Taguchi M. Seto K. Kawahara R. Kawatsu K. Kumeda Y. (2007). Development of a multiplex PCR assay for identification of *Campylobacter coli*, *Campylobacter fetus*, *Campylobacter hyointestinalis* subsp. *hyointestinalis*, *Campylobacter jejuni*, *Campylobacter lari* and *Campylobacter upsaliensis*. J. Med. Microbiol. 56, 1467–1473. 10.1099/jmm.0.47363-0 17965346

[ref153] Yang C. Jiang Y. Huang K. Zhu C. Yin Y. (2003). Application of real-time PCR for quantitative detection of *Campylobacter jejuni* in poultry, milk and environmental water. FEMS Immunol. Med. Microbiol. 38, 265–271. 10.1016/S0928-8244(03)00168-8 14522462

[ref1037] Yang H. Li Y. Johnson M. (2006). Survival and death of *Salmonella* Typhimurium and *Campylobacter jejuni* in processing water and chicken skin during poultry scalding and chilling. J. Food Protect. 64, 770–776. 10.4315/0362-028x-64.6.770 11403124

[ref155] Yolken R. H. (1982). Enzyme immunoassays for the detection of infectious antigens in body fluids: current limitations and future prospects. Rev. Infect. Dis. 4, 35–68. 6803327 10.1093/clinids/4.1.35

[ref154] Yolken R. H. (1988). Nucleic acids or immunoglobulins: which are the molecular probes of the future? Mol. Cell. Probes 2, 87–96. 10.1016/0890-8508(88)90030-8, PMID: 3050454

[ref156] Zeng D. Chen Z. Jiang Y. Xue F. Li B. (2016). Advances and challenges in viability detection of foodborne pathogens. Front. Microbiol. 7, 1–12. 10.3389/fmicb.2016.01833, PMID: 27920757 PMC5118415

[ref157] Zhao C. Ge B. Villena J. D. Sudler R. Yeh E. Zhao S. (2001). Prevalence of *Campylobacter* spp., *Escherichia coli*, and *Salmonella* serovars in retail chicken, turkey, pork, and beef from the greater Washington, D.C., area. Appl. Envrion. Microbiol. 67, 5431–5436. 10.1128/AEM.67.12.5431-5436.2001 PMC93326 11722889

[ref158] Zhao S. Tyson G. H. Chen Y. Li C. Mukherjee S. Young S.. (2016). Whole-genome sequencing analysis accurately predicts antimicrobial resistance phenotypes in *Campylobacter* spp. Appl. Environ. Microbiol. 82, 459–466. 10.1128/AEM.02873-15, PMID: 26519386 PMC4711122

[ref159] Ziprin R. L. Droleskey R. E. Hume M. E. Harvey R. B. (2003). Failure of viable nonculturable *Campylobacter jejuni* to colonize the cecum of newly hatched Leghorn chicks. Avian Dis. 47, 753–758. 10.1637/7015, PMID: 14562908

